# Conversion of Polyethylene to High-Yield Fuel Oil at Low Temperatures and Atmospheric Initial Pressure

**DOI:** 10.3390/ijerph20054048

**Published:** 2023-02-24

**Authors:** Yuanjia Zhang, Xueru Chen, Leilei Cheng, Jing Gu, Yulin Xu

**Affiliations:** 1 Guangzhou Institute of Energy Conversion, Chinese Academy of Sciences, Guangzhou 510640, China; 2 School of Energy Science and Engineering, University of Science and Technology of China, Hefei 230026, China; 3 CAS Key Laboratory of Renewable Energy, Guangzhou 510640, China; 4 Guangdong Provincial Key Laboratory of New and Renewable Energy Research and Development, Guangzhou 510640, China

**Keywords:** pyrolysis, phase transition, polyethylene, low-boiling hydrocarbons

## Abstract

The transformation of waste plastics into fuels via energy-efficient and low-cost pyrolysis could incentivize better waste plastic management. Here, we report pressure-induced phase transitions in polyethylene, which continue to heat up without additional heat sources, prompting the thermal cracking of plastics into premium fuel products. When the nitrogen initial pressure is increased from 2 to 21 bar, a monotonically increasing peak temperature is observed (from 428.1 °C to 476.7 °C). At 21 bar pressure under different atmosphere conditions, the temperature change driven by high-pressure helium is lower than that driven by nitrogen or argon, indicating that phase transition is related to the interaction between long-chain hydrocarbons and intercalated high-pressure medium layers. In view of the high cost of high-pressure inert gases, the promotion or inhibition effect of low-boiling hydrocarbons (transitioning into the gaseous state with increasing temperature) on phase transition is explored, and a series of light components are used as phase transition initiators to replace high-pressure inert gases to experiment. The reason that the quantitative conversion of polyethylene to high-quality fuel products is realized through the addition of 1-hexene at a set temperature of 340 °C and the initial atmospheric pressure. This discovery provides a method for recycling plastics by low energy pyrolysis. In addition, we envisage recovering some of the light components after plastic pyrolysis as phase change initiators for the next batch of the process. This method is able to reduce the cost of light hydrocarbons or high-pressure gas insertion, reduce heat input, and improve material and energy utilization.

## 1. Introduction

Plastics, comprising long-chain organic molecules, have assisted in the economic development and establishment of convenient living conditions in the human world over the last decades. Today, annual plastic production is >400 million tones, and at the same time, global solid waste production had grown steadily over the past 50 years [1,2]. In contrast to biomass-based materials, plastics are difficult to degrade by micro-organisms under natural conditions, and the continuous accumulation of plastics in all our surroundings is causing serious soil and water pollution problems. Importantly, many plastic fragments are being found in the oceans, and micro- and nano-plastics can be ingested by aquatic animals, which exerts an uncertain impact on the human food chain and ecosystem health [3,4]. Current repurposing routes for these unwanted waste plastics occurring globally mainly involve landfilling, incineration, mechanical processing, and pyrolysis. Waste plastics entering landfills pollute soil and groundwater environments and do not degrade for hundreds of years [5]. Although incineration can realize energy recovery in the form of heat, this approach should be avoided as much as possible due to its high global warming potential [6]. New plastics are obtained via the mechanical recycling route, whereby sorted plastics are remelted and extruded [7,8,9]. However, this method produces low-quality plastics [6,10,11] and does not achieve the ultimate goal of eliminating plastic generation.

Pyrolysis is a very promising approach for the disposal of waste plastics because they can be converted into value-added starting materials, fuel oils or chemicals through controlled depolymerization. Waste plastic recycling into starting monomers that are polymerizable into plastic products is an ideal circulation method owing to the absence of property loss [10]. However, this method requires excessive energy consumption considering polyolefins, such as polypropylene and polyethylene. Since polyolefin monomers originate from petroleum, it is feasible to convert polyolefins into gasoline- or diesel-range hydrocarbons. Polyethylene is the most widely produced plastic worldwide [12,13] and was chosen as the feedstock in this work. Researchers have analyzed the decomposition temperature of polyethylene. Marcilla et al. [14] observed that chain scission occurred from 360~385 °C in the pyrolysis process of low-density polyethylene (LDPE). Onwudili et al. [15] employed an autoclave to conduct LDPE pyrolysis experiments and found that oil formation started at ~410 °C by investigating the obtained products. The thermal cracking process of polyolefins requires a high energy consumption, including high temperatures and long processing times. Catalytic pyrolysis can generate fuel products or chemicals with a high added value at lower temperatures [16,17]. However, many common catalysts, such as zeolites, result in a high yield of gases and char residues, which reduces the selectivity of targeting liquid oil products [18,19,20]. Moreover, the high costs of catalysts and deactivation agents constitute a shortcoming of the catalytic process, which limits its industrial implementation [21]. Recently, several studies on the catalytic hydrogenolysis of polyethylene using finely designed catalysts have obtained high yields of valuable products under moderate conditions, but a long residence time was necessary (24 h or even days) [18,22,23,24], as was a high energy demand.

In our previous studies [25], a phase transition phenomenon was observed when long-chain hydrocarbons and their base polymers were heated under high-pressure atmospheric conditions. If the carbon chain exceeded a certain length, temperature increase resulted in feedstock depolymerization, even though the set temperature was far lower than the minimum temperature required for cracking. It was verified that this temperature rise process was initiated by pressure-induced phase transition. Polyethylene was completely converted into oil and gaseous products at a set temperature of 340 °C aided by phase transition. At present, to achieve high-pressure conditions, it is usually necessary to fill reactors with inert high-pressure gases, which leads to a higher process cost. In addition, studies on polymer phase transition have been conducted in the temperature range of solid-state transition. Polotsky et al. [26] observed a coil-to-flower transition phenomenon when a single minority linear chain was embedded into a polymer brush comprising arm-grafted stars through theoretical methods, and this transition was able to be triggered by increasing the chain length, brush grafting density or solvent strength. Li et al. [27] reported pressure-induced phase transition in plastic crystals yielding a very notable entropy change driven by a relatively small pressure. Moreover, the addition of a certain solvent not only improved the heat and mass transfer efficiency between plastics and catalysts but also made plastics swell and helped catalysts enter the inner part of plastics for depolymerization. Wang et al. [28] found that acetic acid had the best swelling capacity for cured epoxy resin, more than 56%, while alcohols had a weaker swelling capacity, about 11~21%. Another study by Wang et al. [29] found that the use of solvent to swell the resin is conducive to the reaction of the catalyst into the resin. Although solvents can swell plastics to a certain extent, at present, only oxygen-containing compounds have a better effect of solvent swelling degradation, while C-C polymer is more stable and not easily degraded by solvent swelling. There are currently few studies on the temperature range of liquid-state phase transition in polymers. As an ideal host with a very high compressibility and extensive disordered characteristics, polyethylene was able to undergo a significant phase change during the pyrolysis process, and significant enthalpy changes could be obtained, which could be expected to promote pyrolysis reactions. Although a very large adiabatic temperature change originating from the liquid–liquid phase transition process of long-chain hydrocarbons has been observed in our previous work, there remains a lack of understanding of its mechanism and application.

The pyrolysis process was not well studied based on the change in temperature of the polyethylene phase transition. Here, we report on the liquid phase to liquid phase transition phenomenon in polymeric materials, which results in a large change in temperature in the closed system, resulting in a significant break in the polyethylene chain and the preparation of high-quality fuel products. In this study, polyethylene pyrolysis experiments were carried out under different pressure conditions to understand pressure-induced phase transition. In order to further reveal the underlying mechanism of pressure induced phase transition, we conducted experiments in different atmospheres. Furthermore, considering the high cost of high-pressure noble gases, different phase transition initiators were used to achieve pressure-induced phase transitions. The specific objectives of the current study are: (1) to understand the pyrolysis behavior of polyethylene phase transition under nitrogen pressure; (2) to reveal the temperature change behavior caused by the pressure induced phase transition during polyethylene pyrolysis under different inert atmospheres; and (3) to explore the possibility of using liquid hydrocarbons with a low boiling point to achieve pressure-induced phase transformation and clarify the inhibition or promotion of specific low carbon hydrocarbon additives on the phase transformation process of polyethylene.

## 2. Materials and Methods

### 2.1. Materials

The principal experiments were carried out using LDPE provided by the Sigma-Aldrich Corporation (St. Louis, MO, USA). 1-hexene (99%), octane (98%) and xylene (99%) were all purchased from Meryer. 1-octene (98%) and 1-decene (95%) were both obtained from Macklin (Rochelle, IL, USA). Chloroform-D (D, 99.8% + 0.03% *v/v* TMS) was acquired at Cambridge Isotope Laboratories (Tewksbury, MA, USA). Dichloromethane (≥99.9%, GC Resolv™) was obtained from Fisher Chemicals (Hampton, NH, USA). The above reagents were used as received.

### 2.2. Experimental Setup and Operating Conditions

**Figure 1:**
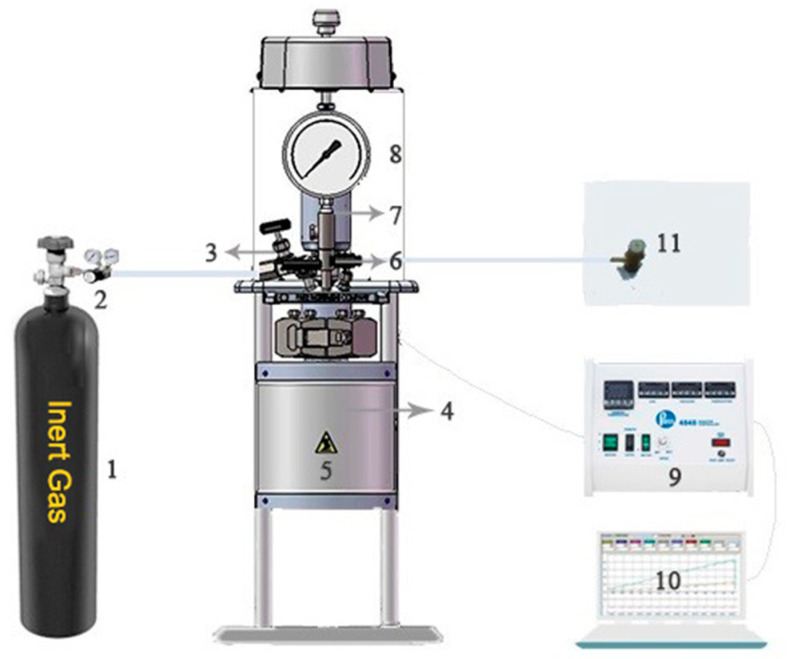
Experimental apparatus used for pyrolysis experiments. 1. Gas cylinder; 2. pressure reducing valve; 3. air inlet valve; 4. autoclave body; 5. heater; 6. air outlet valve; 7. magnetic stirrer; 8. pressure gauge; 9. reaction controller; 10. computer; 11. gas collection bag.

The polyethylene pyrolysis experiments were performed in Parr autoclaves with a magnetic stirrer to mix viscous suspensions. The specific device is shown in Figure 1. Autoclaves mainly comprised four parts: a 100 mL microreactor (Parr 4590), a PID temperature controller, a high-pressure atmosphere supply device and a computer for recording temperature and pressure curves. The pressure value was measured by a pressure sensor. J-type thermocouple and pressure sensor transmitted the temperature and press signals to a computer to obtain a curve of temperature and pressure vs. time. The magnetic stirrer stirred at 200 rpm/min during the test. To ensure an inert atmosphere during the test, the reactor was purged with nitrogen for 20 min before the test started, after that, the inert gas was charged into the reactor body until reaching the set initial pressure value. Then, after closing the inlet valve 3 and outlet valve 6, the reactor was heated to a preset temperature. The heater was removed when the reactor reached peak temperature and the reactor was cooled to room temperature by air. The gaseous product was collected using a gas collection bag, while the liquid product and residual waxes were obtained in the kettle. The pyrolysis conditions of LDPE at different pressures, inert atmospheres (nitrogen, helium and argon) and additives (1-hexene, 1-octene, 1-decene, octane and xylene) are shown in Table 1. Different pressure experiments are denoted as “Px”, where x is the initial pressure value, different atmosphere experiments are denoted as “Ax” and the experiments involving the addition of liquid hydrocarbons are denoted as “Lx”.

### 2.3. Analytical Methods

The gas product was distributed using an Agilent 7890N gas chromatograph equipped with two thermal conductivity detectors (TCD) and a flame ionization detector (FID). Standard gases (including C1-C6 hydrocarbons and hydrogen) were used for calibration, and these volatile compounds were analyzed qualitatively and quantitatively.

The resulting oil product distribution was determined by GC-MS (TRACE 1300ISQ), and the chromatographic conditions were described as follows: (1) a HP-5 capillary column (30 m × 0.25 mm ID × 0.25 µm) was adopted, (2) the injection sample size was 1 μL, (3) helium was used as carrier gas at a flow rate of 0.8 mL/min and (4) GC temperature was kept at 40 °C for 5 min, and then heated to 290 °C at a heating rate of 7 °C/min, followed by maintaining at 290 °C for 10 min; for the MS method, ionization mode EI was adopted, with the ionization energy of 70 eV and ion source temperature of 230 °C. Compared with the NIST 20 MS Spectral library, the chemical components were identified. A semiquantitative method was used to observe the changes in the individual components of the corresponding experiments by calculating the percentage of the chromatographic peak area.

The molecular weight distribution of the resulting liquid/wax product was analyzed on the Agilent PL-GPC 220 equipped with three PL-Gel Hybrid B columns, one PL-Gel Hybrid B Protection Column and one refractive index (RI) detector. Samples were prepared in 1,2,4-trichlorobenzene (TCB), containing 0.01 wt.% 3,5-di-tert-butyl-4-hydroxytoluene (BHT), and heated at 150 °C for at least 1 h. Elution was proceeded using TCB (containing BHT) at a flow rate of 1.0 mL/min at 150 °C. The molecular weight data were calibrated using the Agilent and Polymer Standards Service, Inc. (Palo Alto, CA, USA), monomodal polyethylene standards.

NMR ^1^H and ^13^C NMR were tested using a 500 MHz Bruker spectrometer (^1^H = 500 MHz, ^13^C = 125 MHz). Spectra were analyzed using MestReNova (v14.0.0-23239, Mestrelab Research S. L., A Coruña, Spain). Chemical offsets (δ, ppm) for 1H and 13C were calibrated using the internal solvent signals and referenced to TMS. The branching of liquid products was calculated by 1H NMR using the following formula: branching per 100 carbons = (I_CH3_/3)/[(I_CH_ + I_CH2_ + I_CH3_)/2] × 100. I_CH_, I_CH2_ and I_CH3_ refer to the ^1^H NMR integrations for the methine, methylene and methyl signals, respectively [30,31].

## 3. Results

### 3.1. The Effect of Nitrogen Pressure

In general, the cleavage process of C-C bonds in polyethylene entails an endothermic reaction [16,32,33], but the process of temperature increase occurs under certain conditions, especially in an upper high-pressure atmosphere. In previous studies, we employed different long-chain hydrocarbons as pyrolysis feedstocks to demonstrate that this process was driven by phase transition. There are few studies on the liquid-state phase transition of long-chain hydrocarbons or their polymers, but certain studies have reported that the enthalpy changes occurring during the solid-state transition in plastics from the ordered state to the disordered state are much larger than those in the fusion enthalpy [27,34,35] that occurs in regular solids, such as certain metal materials. Due to this feature, specific plastics are usually adopted as solid-state energy storage materials.

To obtain further mechanistic insights, polyethylene pyrolysis experiments were carried out under different nitrogen pressures to observe the temperature changes induced by phase transition. In this series of experiments, the set temperature was 380 °C. Figure S1 shows temperature and pressure vs. time curves of the blank experiment and the experiments under the different pressures. The blank experiment under an initial pressure of 21 bar revealed no thermal runaway process, and instrument factors could therefore be preliminarily excluded. When 11 g LDPE (M_w_ = 5.412 × 10^3^, Đ = 2.64) was applied as the reactant under an initial pressure of 1 atm, with the other conditions remaining constant (Table 1, P1), the temperature exhibited a higher increase rate after ~305 °C, but the temperature never exceeded the set point of 380 °C (Figure S1b). Interestingly, in the P2 test, the temperature exceeded the set value and reached 428.1 °C. Before the set temperature of 380 °C was reached, due to the high heating rate, the heating switch was automatically deactivated. Thus, the temperature rise occurred due to the internal reaction system. When the initial pressure was increased from 2 to 21 bar, a monotonically increasing peak temperature was observed (from 428.1 °C to 476.7 °C; Figure S1c,j). Phase transition released an enormous amount of heat, which could be compared based on the determined adiabatic temperature changes, as the same initial temperature was applied across all tests. The reason for the temperature runaway phenomenon may be that the increase in pressure increases the phase transition temperature of the reactants, resulting in a liquefaction exothermic phenomenon, while the reaction substrate gathers at the bottom of the reactor under high pressure. The increase in the concentration of the reaction substrate may also be conducive to the occurrence of the exothermic reaction of the free radical polymerization of small and medium molecules during the pyrolysis process. In addition, increasing the temperature resulted in further depolymerization, and the heat release level was able to be indirectly measured through the cracking degree of polyethylene (i.e., the molecular mass of the obtained products). The phase transition process is highly sensitive to the applied pressure, which suggests that a relatively low pressure can drive a highly notable conformation transformation and molecular potential change in long-chain hydrocarbons.

**Figure 2:**
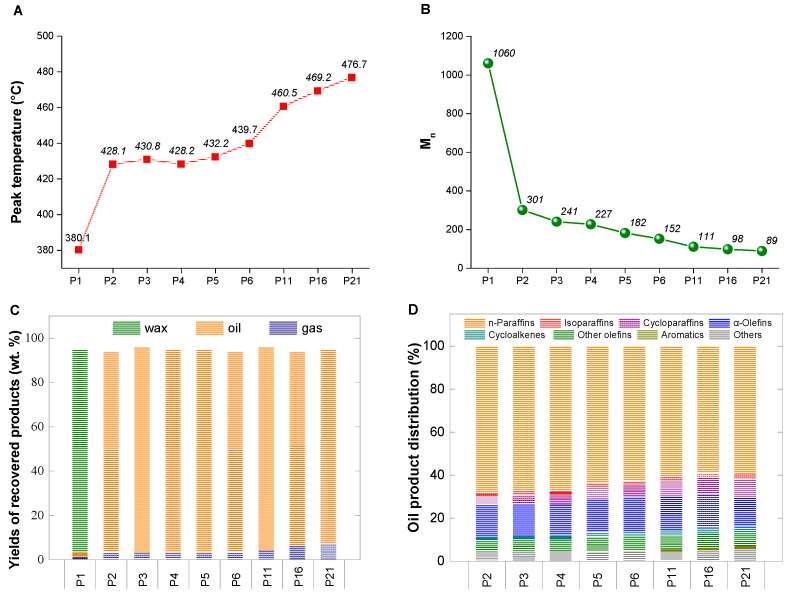
Pyrolysis experiments under different pressure conditions, in nitrogen atmosphere, at switch II and a set temperature of 380 °C. (**A**) Peak temperatures from different pressure experiments. (**B**) Mn of the recovered oil or wax products from different pressure experiments. (**C**) Yields of the collected gas, liquid and wax fractions from different pressure experiments. (**D**) Oil product distribution from different pressure experiments; “others” refer to those components that are difficult to accurately determine.

Although these experiments experienced the same set temperature, the distinct peak temperatures yielded different cracking degrees of polyethylene. The molecular weight distribution and chemical composition of the recovered liquid products were analyzed through gel permeation chromatography (GPC), GC-MS and NMR, which, jointly considered, provide a precise description of the hydrocarbon distribution. First, we compared the peak temperatures of the different tests to the molecular weights of the obtained liquid or wax products (Figure 2A,B). As the initial pressure was increased from 1 atm to 21 bar, the Mn value of the major products decreased from 1060 to 89, indicating that a higher temperature rise resulted in a smaller molecular mass distributions. This may be because the reaction temperature gradually increased as the nitrogen pressure increased, and higher temperatures facilitate the breaking of the C-C bond in polyethylene to form the smaller molecules of light hydrocarbons. The yields of the collected gas, liquid and wax fractions are shown in Figure 2C. Owing to the structural limitations of the autoclave, a small fraction of generated waxes was volatilized and congealed in the upper pipes during the reaction process, which was difficult to recover and is considered to be missing mass. In the P1 test, the recovered wax represented the largest fraction (92 wt.%) of the original polyethylene mass due to its low reaction temperature of 380.1 °C. Under the initial pressures of 2 to 21 bar, oil products accounted for most of the largest fraction of the original polyethylene mass (up to ~90 wt.%). The analysis of gaseous products by GC revealed that with the increase in pressure, the increase in gaseous olefin molecules was less than that of alkane molecules (Table 2). In addition, the CPG analysis of oil products indicated that the molecular weight distribution of the condensed-phase product decreased with increasing pressure, in accordance with the GC-MS results (Table 3). In effect, due to the fixed H/C ratio, the proportion of unsaturated hydrocarbons increased with the degree of polyethylene cracking [15,25], as illustrated in Figure 2D. Particularly, contrary to the atmospheric pressure experiments, the olefin content was reduced, which was confirmed by ^1^H NMR (Table S1), even though a higher peak temperature has led to a high degree of chain rupture, suggesting the occurrence of cycling, polymerization, or hydrogenation reactions, thus consuming olefins or their precursors in the high-pressure pyrolysis process of polyethylene [36,37,38,39].

### 3.2. Effect of the Different Atmospheres

**Figure 3:**
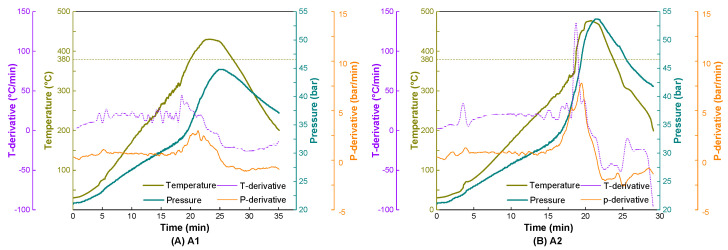
Temperature and pressure vs. time curves of experimental processes in different high-pressure atmospheres (helium, argon and nitrogen).

To explore the influence of reactant phase transition on reaction processes, other inert gases, such as helium and argon, were also screened in comparative experiments under the same conditions as those in the P21 test. As shown in Figure 3, A2 behaved similarly to P21, with a peak temperature of 477.4 °C, which resulted in a liquid product of low molecular weight (Mn = 104, Đ = 3.15). However, both the ramp rate and peak temperature in A1 during the period of increasing temperature were much lower than those in the tests involving the other two gases, although the thermal conductivity of helium is higher than that of nitrogen and argon. Accordingly, the longer-chain molecules of the liquid products (Mn = 220, Đ = 3.57) were obtained in the case of helium, which was also confirmed via GC-MS (Figure S4a).

Sirota et al. [40] explored the structural effects of three high-pressure inert gases on the rotator phases of long-chain alkanes and observed that nitrogen and argon could become intercalated between layers, thus influencing the in-plane coupling of the rotator phases, while helium mainly functioned as a noninteracting pressurized medium. The different behaviors of helium and the other gases preliminarily indicate that phase transition is affected by the interaction of long-chain molecules and the intercalated molecules of the phase change initiator, resulting in varying degrees of conformational changes.

### 3.3. Promotion or Inhibition Effect of Low-Boiling Hydrocarbons on Pyrolysis

Given the high cost of high-pressure inert gases, we employed a low-boiling point liquid to establish a high-pressure atmosphere. The first goal was to identify a reagent that would (a) become a gas with increasing temperature rather than mixing with polyethylene reactants and (b) only contained carbon and hydrogen elements to avoid complicating the product. Liquid short-chain hydrocarbons were applied instead of high-pressure inert gases to achieve the necessary phase behavior. When a mixture comprising 8 g LDPE and 8 g 1-hexene was used as the reactant at a set temperature of 380 °C and a nitrogen pressure of 1 atm, an appreciable peak temperature (463.2 °C; Figure S5a) and a similar low-molecular-weight product distribution were achieved (Figure S6a). These results indicated that the necessary phase conditions could be achieved via more efficient methods. Moreover, on one hand, the liquid hydrocarbon added before volatilization may promote the heat transport of solid polymer materials [18,41]. One the other hand, based on the theory of ‘‘like dissolves like’, the shape similarity between polar liquid hydrocarbons and HDPE was able to promote the diffusion of large PE oligomer molecules in the solvent, contributing to the swelling and solvent depolymerization of LDPE [42].

**Figure 4:**
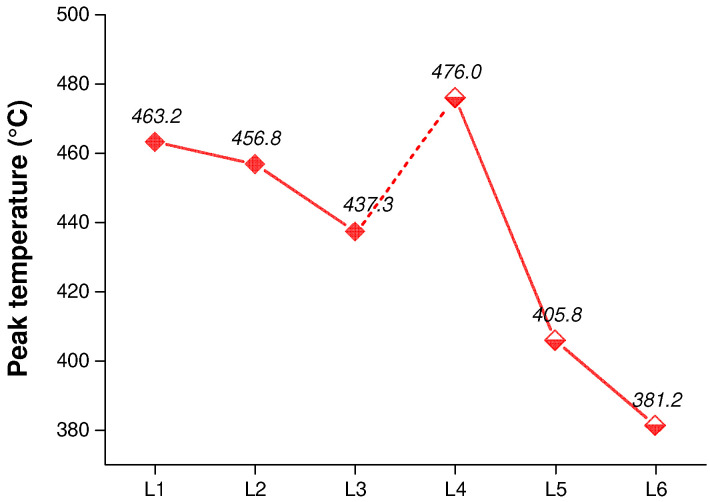
Peak temperatures from LDPE thermal degradation experiments with olefin additives (1-hexene, 1-octene or 1-decene) conducted under an initial atmospheric pressure and high-pressure (21 bar) conditions, respectively, all in a nitrogen atmosphere, at switch II and a set temperature of 380 °C.

Next, to gain further insights into the resultant phase behavior and determine the types of liquids that could be adopted as a phase change initiator, experiments (Table 1, experiments L2, L3, L4, L5 and L6) were conducted involving the addition of a reagent, such as 1-hexene, 1-octene and 1-decene, under atmospheric pressure and a high pressure of 21 bar to observe the differences in reaction processes and products. A phase transition can be expected if low-boiling liquids are added that are transformed into high-pressure gases rather than become blended with the working materials during heating. In contrast to L1, when using 1-octene and 1-decene, the peak temperature decreased to 456.8 °C and 437.3 °C (Figure 4), respectively, resulting in longer-chain liquids (Figure S6b and Figure S6c, respectively). Higher-boiling hydrocarbons are more likely to mix with the reactants and intermediates during the reaction process, and the phase conditions are reduced. Compared to heavier hydrocarbons, small molecules could help establish more satisfactory phase conditions in the pyrolysis process. Thus, recycling a portion of the light products in the next batch to serve as a high-pressure atmosphere at elevated temperatures could lead to a similar temperature change by influencing the phase behavior of the interior system. In the case of L4, when 8 g LDPE and 3 g 1-hexene were applied as starting materials under an initial pressure of 21 bar, a high peak temperature of 476.0 °C was achieved. A slight temperature change was observed when applying 1-octene (experiment L5), with a peak temperature of 405.8 °C. However, the temperature in L6 did not exceed the set point because polyethylene and 1-decene became mixed during the heating period, thus inhibiting the phase transition in the polymeric materials. Again, the results revealed that phase behavior is a critical issue of this process.

To explore the effects of liquid hydrocarbons with different molecular structures on the reaction paths, experiments using hydrocarbon additives (octane or xylene) with similar boiling points, but different bond types were then performed under initial atmospheric and high-pressure conditions (experiments L7, L8, L9 and L10 in Table 1), and these experiments were compared to the aforementioned experiments involving olefin addition. Regardless of the pressure conditions, the peak temperature (435.4 °C and 382.6 °C, as shown in Figure S5g and Figure S5i, respectively) in the test with the octane additive was much lower than that in the test with the 1-octene additive but similar to that in the 1-decene additive test. However, a comparable peak temperature was reached (458.3 °C and 397.0 °C, as shown in Figure S5h and Figure S5j, respectively) in the xylene additive test that reached in the 1-octene additive test in both groups, suggesting that the different chemical constructs of the selected additives did not constitute an important factor influencing this process. Heat release is mainly related to the phase transition heat release of long chain hydrocarbons and phase transition initiators during the heating process and the homogeneous reaction of the reactants at the bottom of the reactor. On the basis of the obtained product characteristics, it was found that the small-molecule hydrocarbons with different chemical compositions applied in this study did not affect the reaction paths because these hydrocarbons were hardly activated, even at the peak temperatures [43].

Finally, based on the aforementioned rules, a pyrolysis experiment with 1-hexene as the phase change initiator was conducted at a set temperature of 340 °C and initial atmospheric pressure (experiment L11, Table 1). Encouragingly, the peak temperature in this experiment reached 445.6 °C (Figure S5k), resulting in a low molecular weight distribution of the recovered liquid product (Figure S6g). When using low-boiling hydrocarbons as phase change initiators, phase transition is achieved even under the initial atmospheric pressure, which avoids the application of high-cost inert gases. Moreover, the external heating time is very short, and low-boiling hydrocarbons can be sourced from the previous batch of pyrolysis products, thus further reducing the processing cost.

## 4. Environmental and Health Issues in Plastic Waste Management

Today, plastic is widely used in society. The global production of plastic has doubled from that of 20 years ago [44]. However, while this number keeps increasing, the management of plastic waste has not developed at the rate proportionate to production, resulting in the accumulation of non-degradable plastic waste. Only 9% of plastic waste is successfully recycled, with most ending up in landfills, incinerators and unmanaged landfills or oceans [2]. This has serious implications for biological health and the Earth’s environment.

At present, the management strategies of plastic upgrading cycles can be divided into energy recovery and material recovery. The former recycles heat energy through incineration, but this method still produces a large amount of polluting gas and is not friendly to the environment. Material recovery can be divided into physical recovery and chemical recovery. The former recycles plastics by breaking them into small particles, but this affects the physical properties of materials. The latter, namely chemical recycling, can degrade plastics into plastic monomer recycling but can also transform plastics into products with higher economic value for upcycling [45]. Scientists are now working to manage waste plastics through chemical upcycling.

This study provides a potential strategy for the chemical upgrade management of waste plastics treated with pressure-induced phase transitions. This method can reduce the cost of light hydrocarbons or high pressure gas insertion, reduce heat input and improve the utilization rate of matter and energy in plastic recycling.

## 5. Conclusions

In summary, we observed a temperature change driven by pressure-induced liquid-state phase transition in the pyrolysis process of polyethylene, and the temperature increase led to more notable depolymerization even at a low set temperature. Highly notable temperature changes were obtained driven by relatively low pressures, and heat input was ceased when the set temperature was reached. Therefore, the external heating time was short. Compared to the traditional thermal treatment of polyethylene, this work achieved a high energy efficiency of polyethylene pyrolysis aided by phase transition in the absence of a catalyst. In this study, low-boiling hydrocarbons and high-pressure inert gases were chosen as phase transition initiators. With the increasing pressure of the inert gases, lower molecular mass fragments were generated as a result of the higher peak temperatures. Moreover, high-pressure nitrogen and argon led to a higher temperature change than that achieved with helium, indicating that the intercalation of gaseous molecules played a crucial role in the phase transition process. A mixture of polyethylene and low-boiling hydrocarbons that transition into the gaseous state at high temperatures could also realize phase transition, thus avoiding the use of high-pressure inert gases. In the case of 1-hexene, the temperature change during the polyethylene pyrolysis process was higher than that in the case of 1-octene or 1-decene. However, the longer-chain liquid hydrocarbons became blended with polyethylene under high pressures, which inhibited the transition process. The conversion of polyethylene into fuel oil with a ~90% yield at a set temperature of 340 °C under the initial atmospheric pressure was realized through the addition of 1-hexene.

Additionally, we speculate that phase transition can be realized at a lower cost via other more efficient approaches, including reactor and process improvement. For instance, recycling heated low-boiling products to serve as a high-pressure medium in the next process batch could reduce the cost of light hydrocarbons or high-pressure inert gases and reduce heat input, which necessitates a corresponding connection between reactor and process control measures. Although the aforementioned experiments were conducted from different perspectives, the microscopic origin of phase transition, such as conformational energetics and anharmonic lattice dynamics, has not been precisely described. Efforts are currently underway to explore the microscopic phase transition mechanism in long-chain hydrocarbons and their polymers via in situ measurement methods. Furthermore, this novel discovery is of vital importance to other fields, such as phase transition in polymers, oil and gas genesis and energy storage materials.

## Figures and Tables

**Table 1:** Different conditional experiments of LDPE pyrolysis by varying the pressure; inert atmospheres, such as nitrogen, helium and argon; and additives, such as 1-hexene, 1-octene, 1-decene, octane and xylene.

Name	Reactants	Reactant Mass (g)	Set Temperature (°C)	Initial Pressure (Bar)	Atmosphere
Blank	-	-	380	21	Nitrogen
P1	LDPE	11	380	1 atm	Nitrogen
P2	LDPE	11	380	2	Nitrogen
P3	LDPE	11	380	3	Nitrogen
P4	LDPE	11	380	4	Nitrogen
P5	LDPE	11	380	5	Nitrogen
P6	LDPE	11	380	6	Nitrogen
P11	LDPE	11	380	11	Nitrogen
P16	LDPE	11	380	16	Nitrogen
P21	LDPE	11	380	21	Nitrogen
A1	LDPE	11	380	21	Helium
A2	LDPE	11	380	21	Argon
L1	LDPE + 1-hexene	8 + 8	380	1 atm	Nitrogen
L2	LDPE + 1-octene	8 + 8	380	1 atm	Nitrogen
L3	LDPE + 1-decene	8 + 8	380	1 atm	Nitrogen
L4	LDPE + 1-hexene	8 + 3	380	21	Nitrogen
L5	LDPE + 1-octene	8 + 3	380	21	Nitrogen
L6	LDPE + 1-decene	8 + 3	380	21	Nitrogen
L7	LDPE + octane	8 + 8	380	1 atm	Nitrogen
L8	LDPE + xylene	8 + 8	380	1 atm	Nitrogen
L9	LDPE + octane	8 + 3	380	21	Nitrogen
L10	LDPE + xylene	8 + 3	380	21	Nitrogen
L11	LDPE + 1-hexene	8 + 8	340	1 atm	Nitrogen

**Table 2:** Chemical composition of gaseous products from different pressure experiments (listed in Table 1).

Name	Gaseous Components (mg/g LDPE)								
	P1	P2	P3	P4	P5	P6	P11	P16	P21
Hydrogen	0.00	0.00	0.11	0.10	0.15	0.19	0.23	0.25	0.27
Methane	0.21	0.07	2.77	2.89	3.72	4.34	5.55	6.34	6.99
Ethane	0.24	0.28	4.06	4.39	5.15	5.98	8.79	10.34	11.65
Ethylene	0.09	0.09	1.54	1.48	1.70	1.90	2.13	2.03	1.91
Propane	0.30	0.42	4.37	4.87	4.62	5.19	8.68	10.66	11.55
Propene	0.17	0.33	3.83	4.08	3.93	4.35	6.34	6.87	6.68
C4 alkanes	0.15	0.22	1.89	2.27	1.85	2.12	3.57	4.90	4.82
C4 olefins	0.14	0.17	0.19	0.24	1.35	1.47	2.14	2.54	2.24
C5	0.07	0.17	1.05	1.42	1.25	1.67	2.68	3.23	3.63
C6	0.35	0.37	0.77	0.82	1.00	0.47	0.89	1.01	0.94
Sum	1.73	2.12	20.58	22.57	24.72	27.68	41.00	48.18	50.67

**Table 3:** Chemical composition of liquid products from different pressure experiments (listed in Table 1).

Components	Peak Area %							
	P2	P3	P4	P5	P6	P11	P16	P21
**n-paraffins**	68.46	67.37	67.48	63.88	62.76	60.83	59.17	59.56
C4	1.91	1.45	1.34	1.19	1.59	1.08	2.25	0.77
C5	3.86	3.25	3.21	3.14	3.89	3.14	4.81	3.12
C6	3.68	3.28	3.41	3.23	3.64	3.59	4.58	5.98
C7	3.16	3.07	3.29	4.03	4.24	4.52	4.43	4.87
C8	3.05	3.10	3.31	3.83	4.09	4.34	4.44	4.68
C9	2.71	2.85	2.99	3.34	3.61	3.85	3.79	4.08
C10	2.71	2.93	2.94	3.20	3.49	3.66	3.61	3.89
C11	2.70	3.06	2.95	3.17	3.43	3.66	3.48	3.75
C12	2.56	2.93	2.79	3.09	3.30	3.38	3.32	3.61
C13	2.39	2.67	2.57	2.85	2.82	3.02	2.75	3.00
C14	2.24	2.45	2.39	2.53	2.61	2.63	2.50	2.62
C15	2.62	2.86	2.77	2.75	2.79	2.83	2.65	2.74
C15+	34.85	33.47	33.53	27.52	23.26	21.14	16.57	16.46
**Isoparaffins**	1.37	2.13	1.69	1.96	1.73	1.79	2.02	2.38
**Cycloparaffins**	4.05	4.05	4.48	5.48	5.90	7.26	7.89	8.38
Cyclopentane, methyl-	0.44	0.41	0.44	0.58	0.65	0.72	0.89	0.84
Cyclohexane, methyl-	0.75	0.74	0.78	0.95	1.03	1.12	1.22	1.26
Cyclopentane, ethyl-	0.24	0.25	0.26	0.33	0.37	0.41	0.46	0.48
Cyclohexane, ethyl-	0.36	0.36	0.37	0.45	0.50	0.56	0.59	0.63
Others	2.26	2.29	2.62	3.16	3.37	4.44	4.73	5.17
**α-Olefins**	14.99	14.85	14.62	15.41	15.68	15.78	15.50	13.67
C6	2.68	2.41	2.29	2.35	2.56	2.15	2.42	2.06
C7	1.87	1.74	1.80	2.18	2.25	2.19	2.20	2.28
C8	2.08	2.05	2.11	2.35	2.40	2.38	2.27	1.60
C9	1.27	1.22	1.32	1.42	1.52	1.60	1.50	1.29
C10	1.47	1.58	1.50	1.52	1.59	1.34	1.23	1.24
C10+	5.63	5.84	5.60	5.59	5.36	6.12	5.88	5.20
**Cycloalkenes**	1.60	1.64	1.65	2.15	2.49	2.60	2.70	2.77
**Other olefins**	5.27	5.34	5.53	6.90	4.33	6.45	6.56	6.32
**Aromatics**	-	-	-	0.31	0.34	1.19	1.29	1.53
Benzene	-	-	-	-	-	-	-	-
Toluene	-	-	-	0.31	0.34	0.53	0.61	0.75
Xylene	-	-	-	-	-	-	-	-
Other MAHs	-	-	-	-	-	0.65	0.68	0.78
**Others**	4.26	4.63	4.54	3.90	4.33	4.10	4.87	5.40

## Data Availability

Not applicable.

## Supplementary Materials

The following supporting information can be downloaded at: https://www.mdpi.com/article/10.3390/ijerph20054048/s1, Figure S1. Temperature and pressure vs. time curves of different pressure experiments (listed in Table 1). Figure S2. GC-MS chromatograms of the recovered oil samples from different pressure experiments (listed in Table 1). Figure S3. 1H NMR spectra (red lines) and 13C NMR spectra (bule lines) of the oil products obtained from different pressure experiments (listed in Table 1). * and # indicate the solvent (chloroform-D) and TMS, respectively. Figure S4. GC-MS chromatograms of the recovered oil samples from different atmosphere experiments (listed in Table 1). Figure S5. Temperature and pressure vs. time curves of experiments of adding liquid hydrocarbon (1-hexene, 1-octene, 1-decene, 1-octene or xylene) (listed in Table 1). Figure S6. GC-MS chromatograms of the recovered liquid samples from experiments of adding liquid hydrocarbon (1-hexene, 1-octene, 1-decene, 1-octene or xylene) (listed in Table 1). Note that the peak of added liquid hydrocarbon is deducted. Table S1. Different proton types and shift region from ^1^H NMR spectra of liquid products.

## References

[1] Geyer R., Jambeck J.R., Law K.L. (2017). Production, use, and fate of all plastics ever made. Sci. Adv..

[2] Yusuf A.A., Dankwa Ampah J., Soudagar M.E.M., Veza I., Kingsley U., Afrane S., Jin C., Liu H., Elfasakhany A., Buyondo K.A. (2022). Effects of hybrid nanoparticle additives in n-butanol/waste plastic oil/diesel blends on combustion, particulate and gaseous emissions from diesel engine evaluated with entropy-weighted PROMETHEE II and TOPSIS: Environmental and health risks of plastic waste. Energy Convers. Manag..

[3] Weithmann N., Möller J.N., Löder M.G.J., Piehl S., Laforsch C., Freitag R. (2018). Organic fertilizer as a vehicle for the entry of microplastic into the environment. Sci. Adv..

[4] Wang T., Li B., Zou X., Wang Y., Li Y., Xu Y., Mao L., Zhang C., Yu W. (2019). Emission of primary microplastics in mainland China: Invisible but not negligible. Water Res..

[5] Jiao X., Zheng K., Hu Z., Zhu S., Sun Y., Xie Y. (2021). Conversion of Waste Plastics into Value-Added Carbonaceous Fuels under Mild Conditions. Adv. Mater..

[6] Vollmer I., Jenks M.J.F., Roelands M.C.P., White R.J., van Harmelen T., de Wild P., van der Laan G.P., Meirer F., Keurentjes J.T.F., Weckhuysen B.M. (2020). Beyond Mechanical Recycling: Giving New Life to Plastic Waste. Angew. Chem. Int. Ed..

[7] Gu F., Guo J., Zhang W., Summers P.A., Hall P. (2017). From waste plastics to industrial raw materials: A life cycle assessment of mechanical plastic recycling practice based on a real-world case study. Sci. Total Environ..

[8] Kratish Y., Li J., Liu S., Gao Y., Marks T.J. (2020). Polyethylene Terephthalate Deconstruction Catalyzed by a Carbon-Supported Single-Site Molybdenum-Dioxo Complex. Angew. Chem. Int. Ed..

[9] Kumar A., von Wolff N., Rauch M., Zou Y.-Q., Shmul G., Ben-David Y., Leitus G., Avram L., Milstein D. (2020). Hydrogenative Depolymerization of Nylons. J. Am. Chem. Soc..

[10] Coates G.W., Getzler Y. (2020). Chemical recycling to monomer for an ideal, circular polymer economy. Nat. Rev. Mater..

[11] Ragaert K., Delva L., Van Geem K. (2017). Mechanical and chemical recycling of solid plastic waste. Waste Manag..

[12] Zhou C., Fang W., Xu W., Cao A., Wang R. (2014). Characteristics and the recovery potential of plastic wastes obtained from landfill mining. J. Clean. Prod..

[13] Santos E., Rijo B., Lemos F., Lemos M.A.N.D.A. (2019). A catalytic reactive distillation approach to high density polyethylene pyrolysis—Part 1-Light olefin production. Chem. Eng. J..

[14] Marcilla A., Beltrán M.I., Navarro R. (2009). Evolution of products during the degradation of polyethylene in a batch reactor. J. Anal. Appl. Pyrolysis.

[15] Onwudili J.A., Insura N., Williams P.T. (2009). Composition of products from the pyrolysis of polyethylene and polystyrene in a closed batch reactor: Effects of temperature and residence time. J. Anal. Appl. Pyrolysis.

[16] Al-Salem S.M., Antelava A., Constantinou A., Manos G., Dutta A. (2017). A review on thermal and catalytic pyrolysis of plastic solid waste (PSW). J. Environ. Manag..

[17] Sharuddin S.D.A., Abnisa F., Daud W., Aroua M.K. (2016). A review on pyrolysis of plastic wastes. Energy Convers. Manag..

[18] Zhang F., Zeng M., Yappert R.D., Sun J., Lee Y.-H., LaPointe A.M., Peters B., Abu-Omar M.M., Scott S.L. (2020). Polyethylene upcycling to long-chain alkylaromatics by tandem hydrogenolysis/aromatization. Science.

[19] Lee N., Joo J., Lin K.-Y.A., Lee J. (2021). Waste-to-Fuels: Pyrolysis of Low-Density Polyethylene Waste in the Presence of H-ZSM-11. Polymers.

[20] Miandad R., Barakat M.A., Rehan M., Aburiazaiza A.S., Ismail I.M.I., Nizami A.S. (2017). Plastic waste to liquid oil through catalytic pyrolysis using natural and synthetic zeolite catalysts. Waste Manag..

[21] Mullen C.A., Dorado C., Boateng A.A. (2018). Catalytic co-pyrolysis of switchgrass and polyethylene over HZSM-5: Catalyst deactivation and coke formation. J. Anal. Appl. Pyrolysis.

[22] Jia X., Qin C., Friedberger T., Guan Z., Huang Z. (2016). Efficient and selective degradation of polyethylenes into liquid fuels and waxes under mild conditions. Sci. Adv..

[23] Tennakoon A., Wu X., Paterson A.L., Patnaik S., Pei Y., LaPointe A.M., Ammal S.C., Hackler R.A., Heyden A., Slowing I.I. (2020). Catalytic upcycling of high-density polyethylene via a processive mechanism. Nat. Catal..

[24] Celik G., Kennedy R.M., Hackler R.A., Ferrandon M., Tennakoon A., Patnaik S., LaPointe A.M., Ammal S.C., Heyden A., Perras F.A. (2019). Upcycling Single-Use Polyethylene into High-Quality Liquid Products. ACS Cent. Sci..

[25] Cheng L., Gu J., Wang Y., Zhang J., Yuan H., Chen Y. (2020). Polyethylene high-pressure pyrolysis: Better product distribution and process mechanism analysis. Chem. Eng. J..

[26] Polotsky A.A., Kazakov A.D., Birshtein T.M. (2017). Linear minority chain in a star brush: The coil-to-flower transition. Polymer.

[27] Li B., Kawakita Y., Ohira-Kawamura S., Sugahara T., Wang H., Wang J., Chen Y., Kawaguchi S.I., Kawaguchi S., Ohara K. (2019). Colossal barocaloric effects in plastic crystals. Nature.

[28] Wang Y., Cui X., Ge H., Yang Y., Wang Y., Zhang C., Li J., Deng T., Qin Z., Hou X. (2015). Chemical Recycling of Carbon Fiber Reinforced Epoxy Resin Composites via Selective Cleavage of the Carbon–Nitrogen Bond. ACS Sustain. Chem. Eng..

[29] Wang Y., Cui X., Yang Q., Deng T., Wang Y., Yang Y., Jia S., Qin Z., Hou X. (2015). Chemical recycling of unsaturated polyester resin and its composites via selective cleavage of the ester bond. Green Chem..

[30] Sadykov B.R., Starikov V.P., Sadykov R.K., Kalabin G.A. (2012). Determination of the fractional composition of merchantable oil using quantitative 1 H NMR spectra. Pet. Chem..

[31] Morgenstern M., Cline J., Meyer S., Cataldo S. (2006). Determination of the Kinetics of Biodiesel Production Using Proton Nuclear Magnetic Resonance Spectroscopy (1H NMR). Energy Fuels.

[32] Hu Q., Tang Z., Yao D., Yang H., Shao J., Chen H. (2020). Thermal behavior, kinetics and gas evolution characteristics for the co-pyrolysis of real-world plastic and tyre wastes. J. Clean. Prod..

[33] Mazloum S., Awad S., Allam N., Aboumsallem Y., Loubar K., Tazerout M. (2021). Modelling plastic heating and melting in a semi-batch pyrolysis reactor. Appl. Energy.

[34] Breed L., Murrill E. (1970). Solid-solid phase transitions determined by differential scanning calorimetry: Part I. Tetrahedral substances. Thermochim. Acta.

[35] Chandra D., Ding W., Lynch R.A., Tomilinson J.J. (1991). Phase transitions in “plastic crystals”. J. Less Common Met..

[36] Zhang Y., Duan D., Lei H., Villota E., Ruan R. (2019). Jet fuel production from waste plastics via catalytic pyrolysis with activated carbons. Appl. Energy.

[37] Zhao D., Wang X., Miller J.B., Huber G.W. (2020). The Chemistry and Kinetics of Polyethylene Pyrolysis: A Process to Produce Fuels and Chemicals. ChemSusChem.

[38] Kumari A., Kumar S. (2017). Pyrolytic degradation of polyethylene in autoclave under high pressure to obtain fuel. J. Anal. Appl. Pyrolysis.

[39] Ding K., Liu S., Huang Y., Liu S., Zhou N., Peng P., Wang Y., Chen P., Ruan R. (2019). Catalytic microwave-assisted pyrolysis of plastic waste over NiO and HY for gasoline-range hydrocarbons production. Energy Convers. Manag..

[40] Sirota E.B., Singer D.M., King H.E. (1994). Structural effects of high pressure gas on the rotator phases of normal alkanes. J. Chem. Phys..

[41] Mohanraj C., Senthilkumar T., Chandrasekar M. (2017). A review on conversion techniques of liquid fuel from waste plastic materials. Int. J. Energy Res..

[42] Jia C., Xie S., Zhang W., Intan N.N., Sampath J., Pfaendtner J., Lin H. (2021). Deconstruction of high-density polyethylene into liquid hydrocarbon fuels and lubricants by hydrogenolysis over Ru catalyst. Chem Catal..

[43] Shi S. (2018). Advances in modeling hydrocarbon cracking kinetic predictions by quantum chemical theory: A review. Int. J. Energy Res..

[44] Jehanno C., Alty J.W., Roosen M., De Meester S., Dove A.P., Chen E.Y., Leibfarth F.A., Sardon H. (2022). Critical advances and future opportunities in upcycling commodity polymers. Nature.

[45] Chen H., Wan K., Zhang Y., Wang Y. (2021). Waste to Wealth: Chemical Recycling and Chemical Upcycling of Waste Plastics for a Great Future. ChemSusChem.

