# Supplementary material for: Conversion of Polyethylene to High-Yield Fuel Oil at Low Temperatures and Atmospheric Initial Pressure
Source: Int J Environ Res Public Health. 2023 Feb 24;20(5):4048. doi: 10.3390/ijerph20054048 (PMC10001737; doi:10.3390/ijerph20054048)
Supplement: Supplementary file 1 [file ijerph-20-04048-s001.zip › ijerph-2220616-supplementary.pdf]

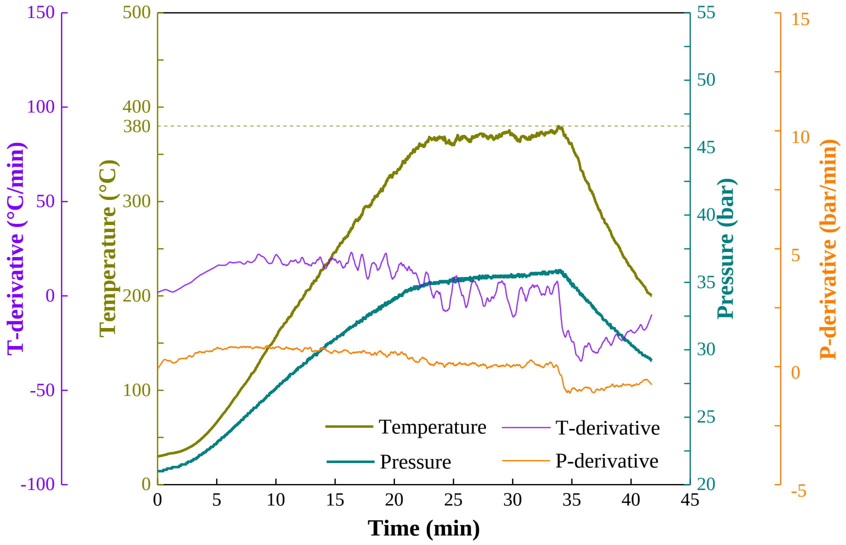

(a) Blank

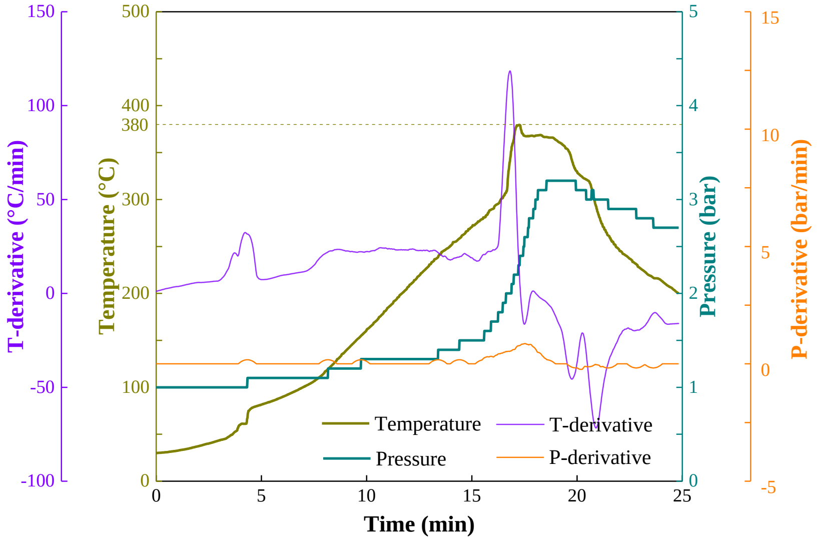

(b) P1

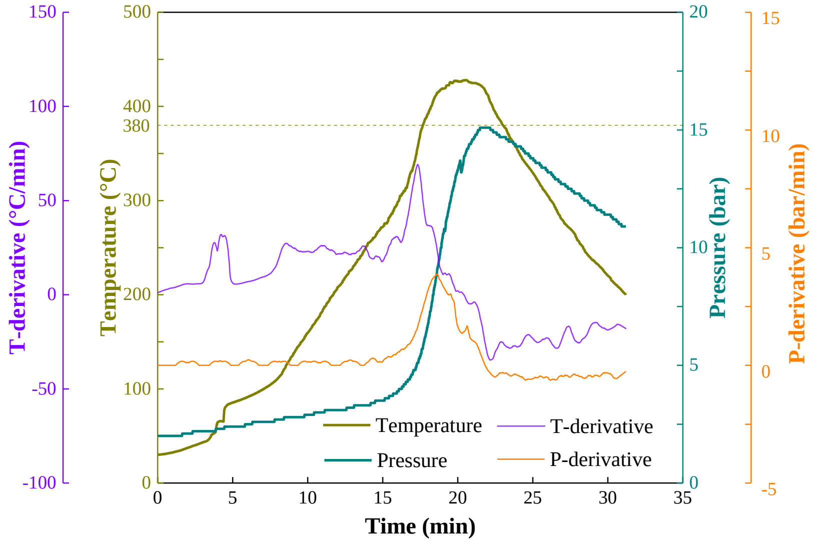

(c) P2

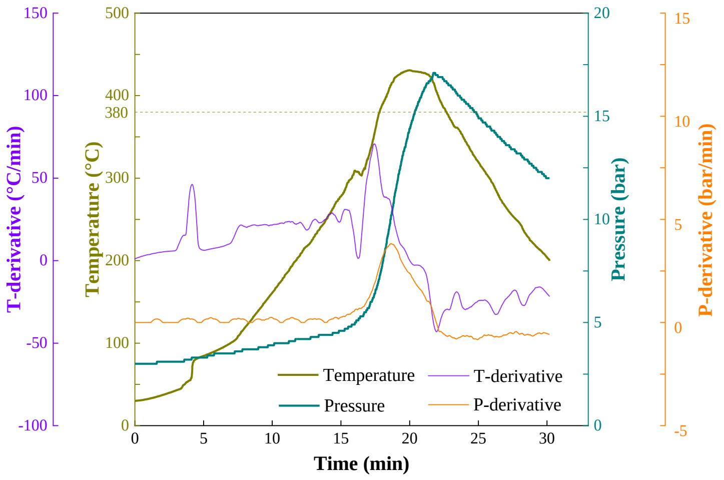

T-derivative (°C/min)
Temperature (°C)
Pressure (bar)
P-derivative (bar/min)
Time (min)
Temperature
Pressure
T-derivative
P-derivative
380

(d) P3

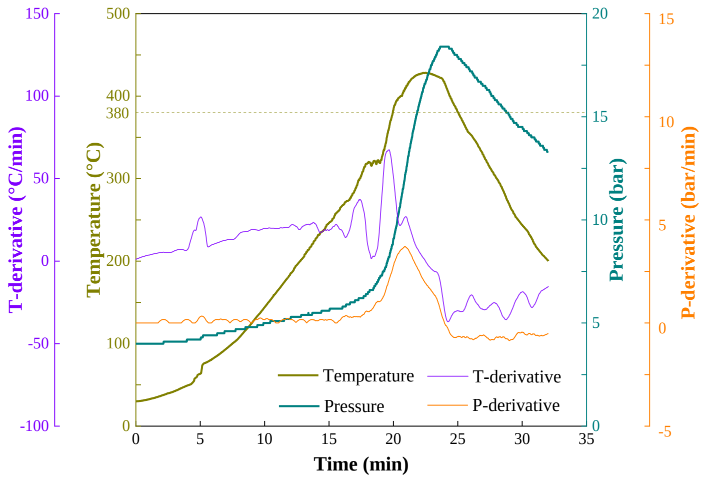

T-derivative (°C/min)
Temperature (°C)
Pressure (bar)
P-derivative (bar/min)
Time (min)
Temperature
Pressure
T-derivative
P-derivative
380

(e) P4

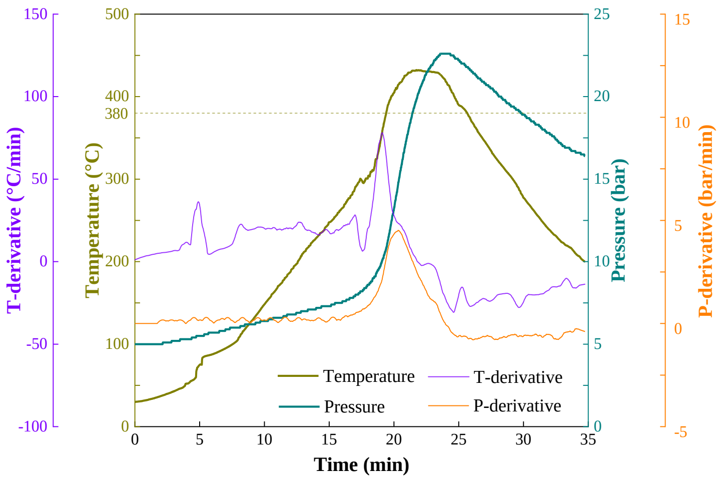

T-derivative (°C/min)
Temperature (°C)
Pressure (bar)
P-derivative (bar/min)
Time (min)
Temperature
Pressure
T-derivative
P-derivative
380

(f) P5

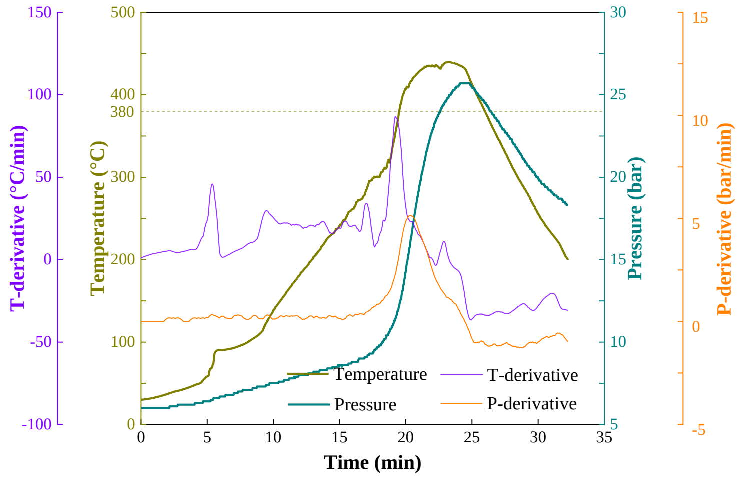

(g) P6

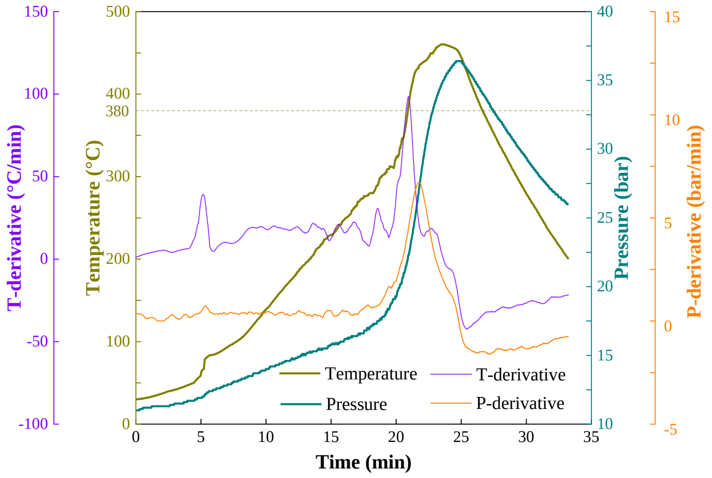

(h) P11

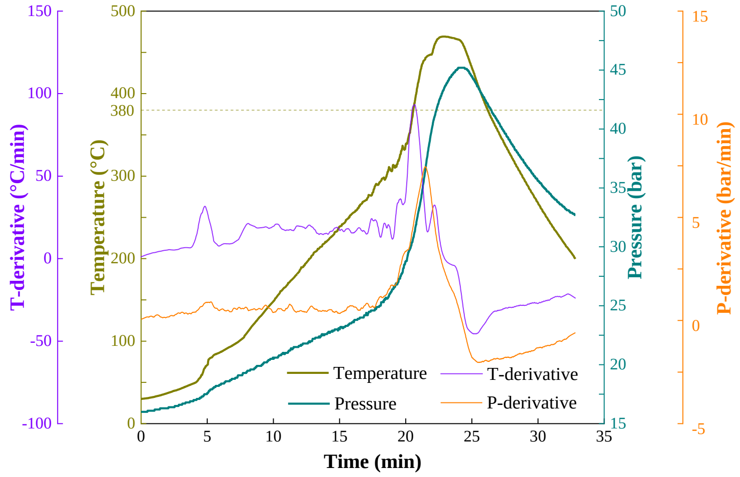

(i) P16

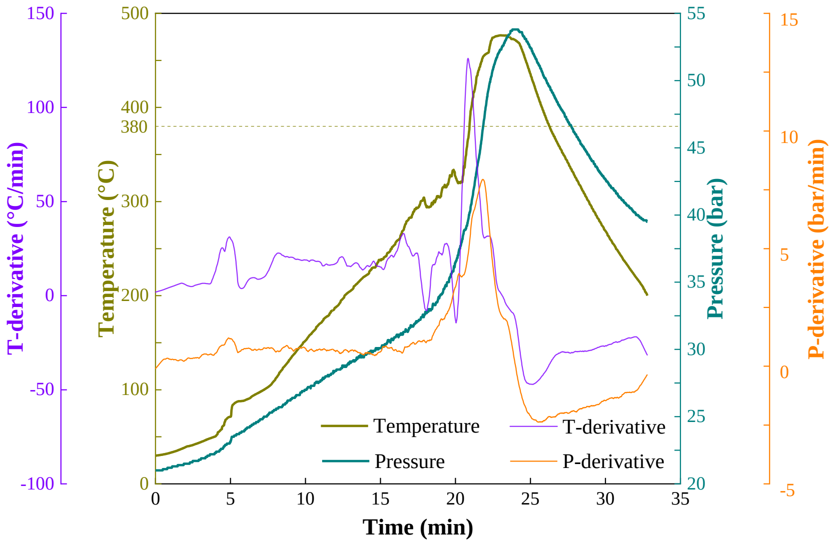

(j) P21

Figure S1. Temperature and pressure vs. time curves of different pressure experiments (listed in Table 1).

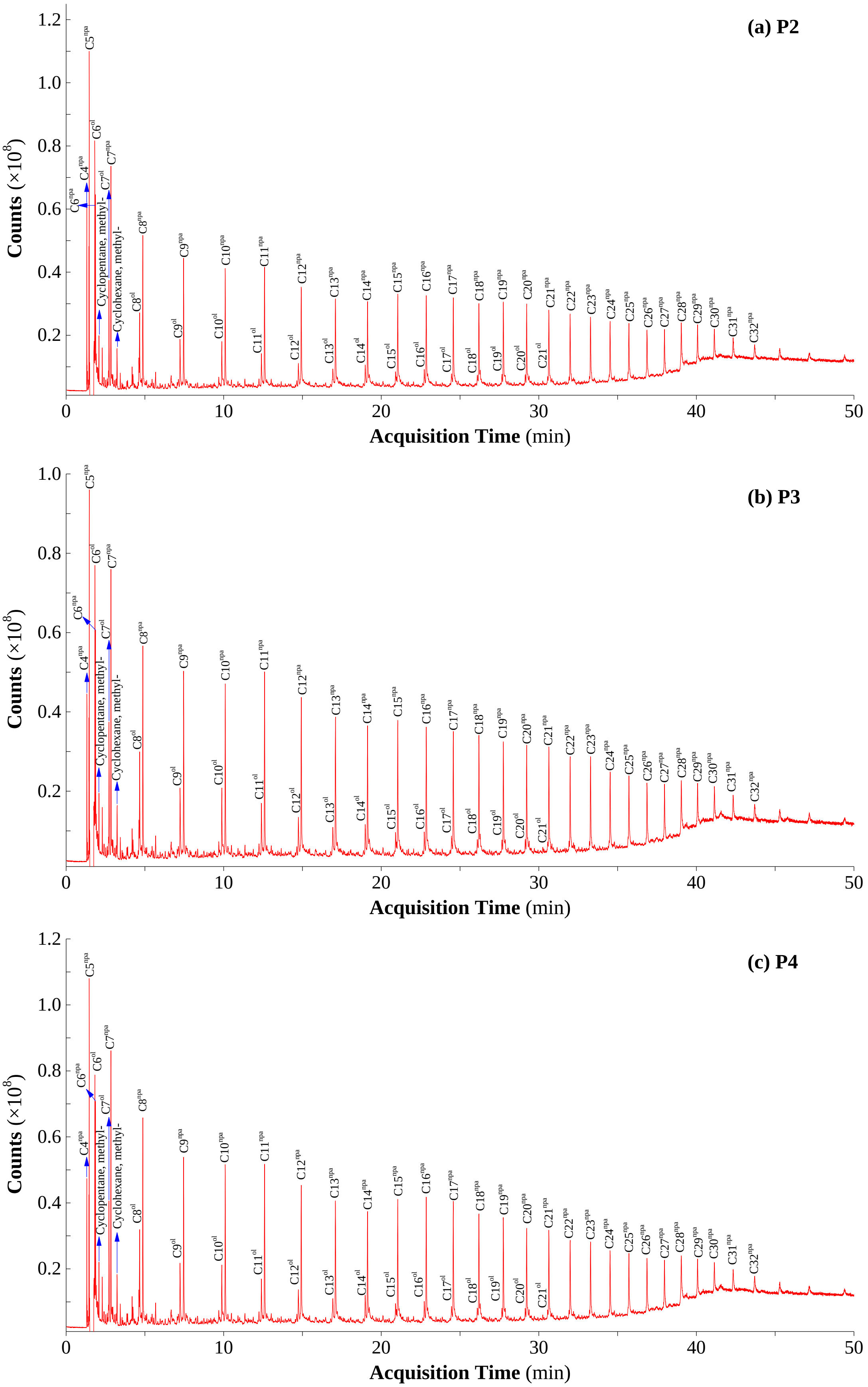
(a) P2
(b) P3
(c) P4
Counts ($\times10^8$)
Acquisition Time (min)
Cyclopentane, methyl-
Cyclohexane, methyl-
$C4^{npa}$ $C5^{npa}$ $C6^{npa}$ $C7^{npa}$ $C8^{npa}$ $C9^{npa}$ $C10^{npa}$ $C11^{npa}$ $C12^{npa}$ $C13^{npa}$ $C14^{npa}$ $C15^{npa}$ $C16^{npa}$ $C17^{npa}$ $C18^{npa}$ $C19^{npa}$ $C20^{npa}$ $C21^{npa}$ $C22^{npa}$ $C23^{npa}$ $C24^{npa}$ $C25^{npa}$ $C26^{npa}$ $C27^{npa}$ $C28^{npa}$ $C29^{npa}$ $C30^{npa}$ $C31^{npa}$ $C32^{npa}$
$C6^{ol}$ $C7^{ol}$ $C8^{ol}$ $C9^{ol}$ $C10^{ol}$ $C11^{ol}$ $C12^{ol}$ $C13^{ol}$ $C14^{ol}$ $C15^{ol}$ $C16^{ol}$ $C17^{ol}$ $C18^{ol}$ $C19^{ol}$ $C20^{ol}$ $C21^{ol}$

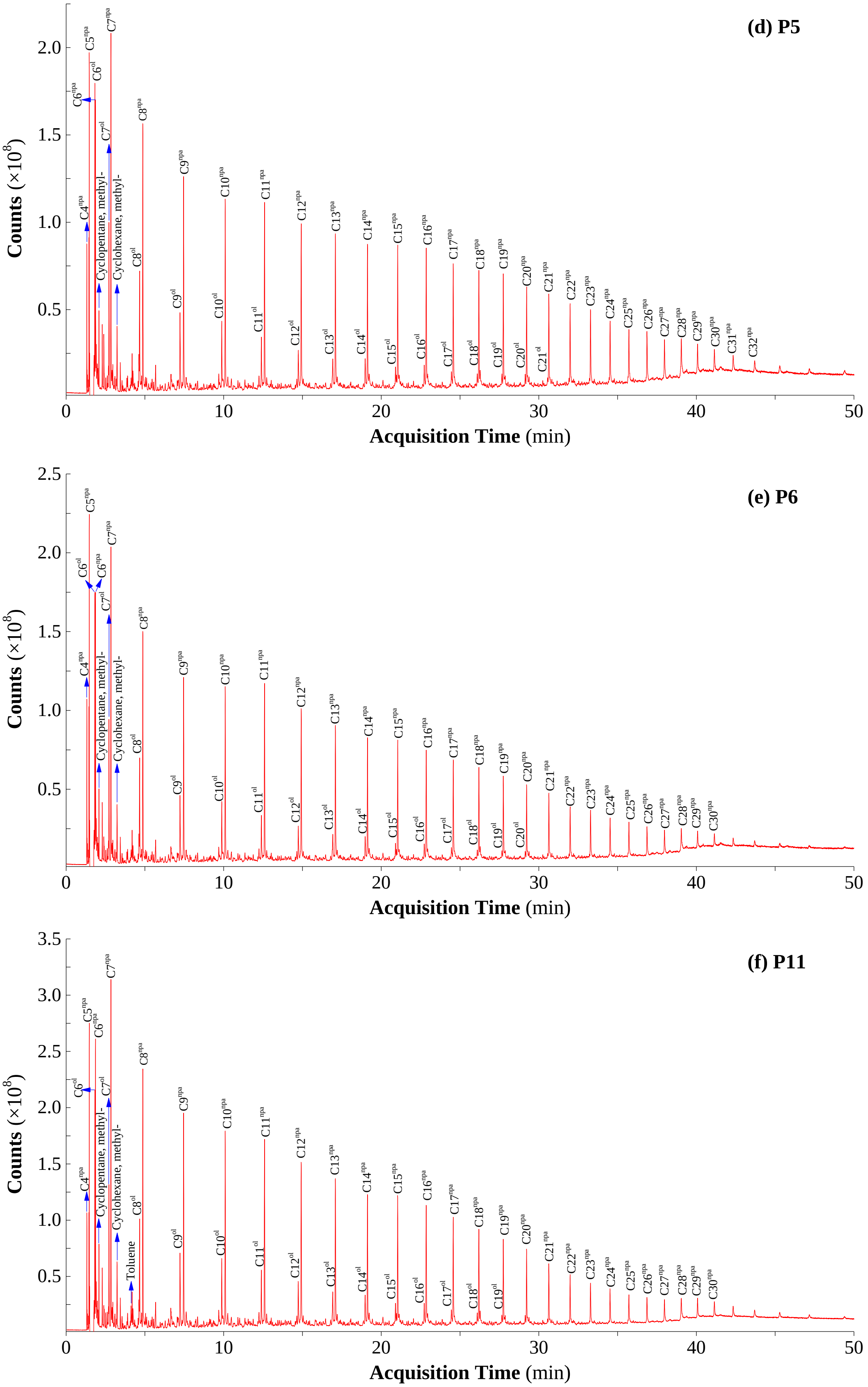

(d) P5
(e) P6
(f) P11
Counts (×10^8)
Acquisition Time (min)
0
10
20
30
40
50
0.5
1.0
1.5
2.0
2.5
3.0
3.5
Cyclopentane, methyl-
Cyclohexane, methyl-
Toluene
C4npa
C5npa
C6npa
C6ol
C7npa
C7ol
C8npa
C8ol
C9npa
C9ol
C10npa
C10ol
C11npa
C11ol
C12npa
C12ol
C13npa
C13ol
C14npa
C14ol
C15npa
C15ol
C16npa
C16ol
C17npa
C17ol
C18npa
C18ol
C19npa
C19ol
C20npa
C20ol
C21npa
C21ol
C22npa
C23npa
C24npa
C25npa
C26npa
C27npa
C28npa
C29npa
C30npa
C31npa
C32npa

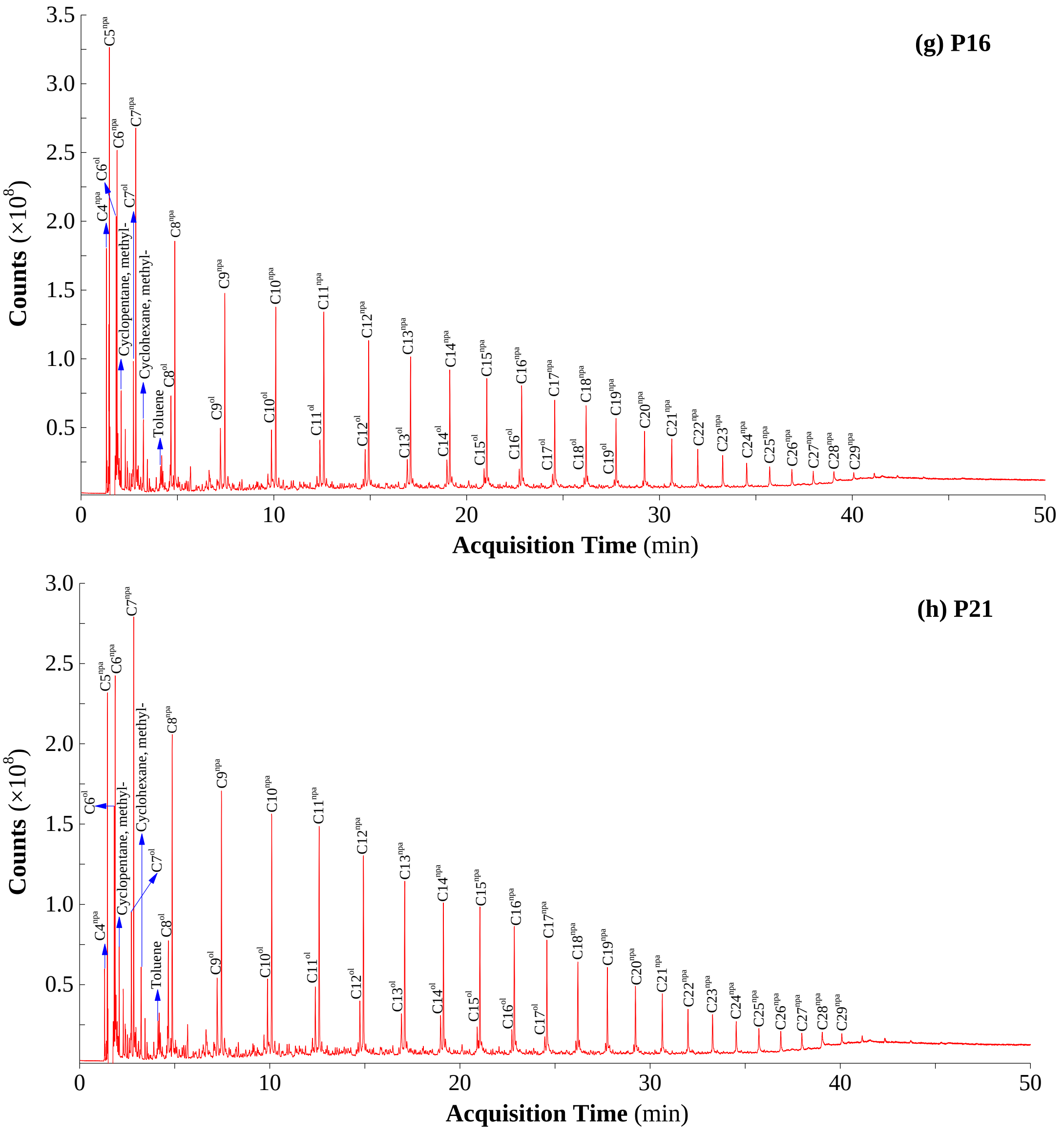

Figure S2. GC-MS chromatograms of the recovered oil samples from different pressure experiments (listed in Table 1).

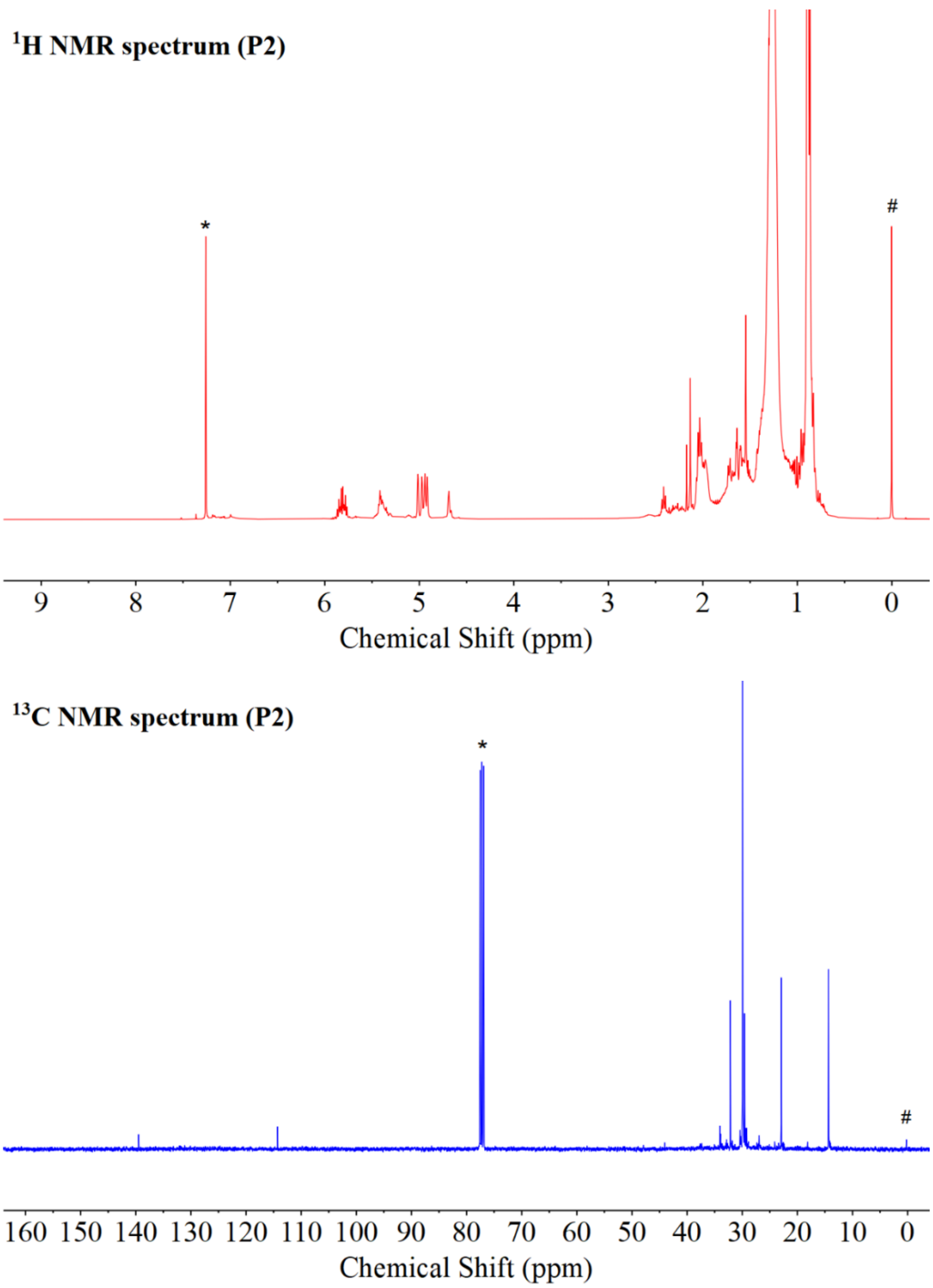

^{1}H NMR spectrum (P2)
*
#
9 8 7 6 5 4 3 2 1 0
Chemical Shift (ppm)
^{13}C NMR spectrum (P2)
*
#
160 150 140 130 120 110 100 90 80 70 60 50 40 30 20 10 0
Chemical Shift (ppm)

(a)

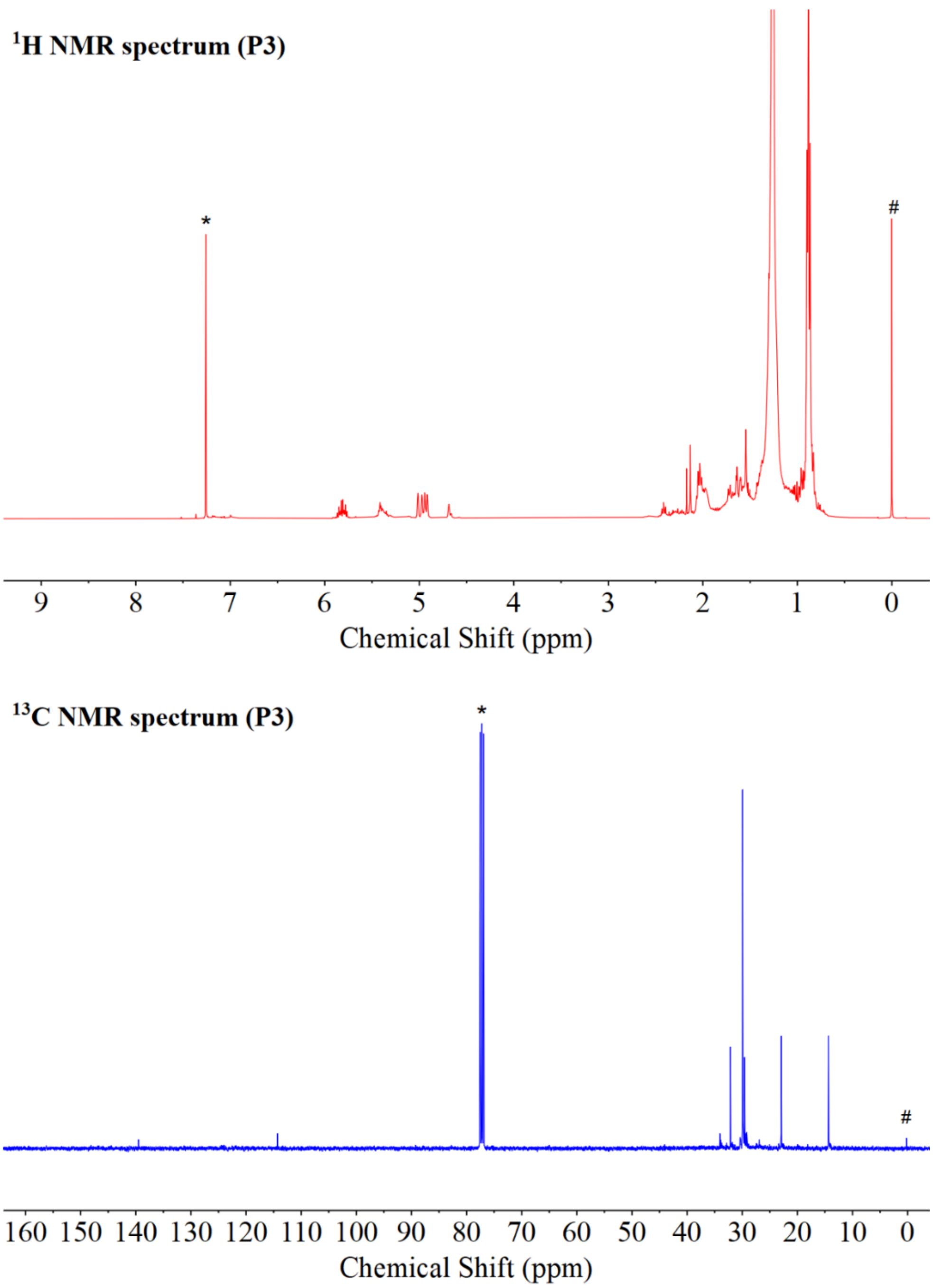

1H NMR spectrum (P3)
*
#
9 8 7 6 5 4 3 2 1 0
Chemical Shift (ppm)
13C NMR spectrum (P3)
*
#
160 150 140 130 120 110 100 90 80 70 60 50 40 30 20 10 0
Chemical Shift (ppm)

(b)

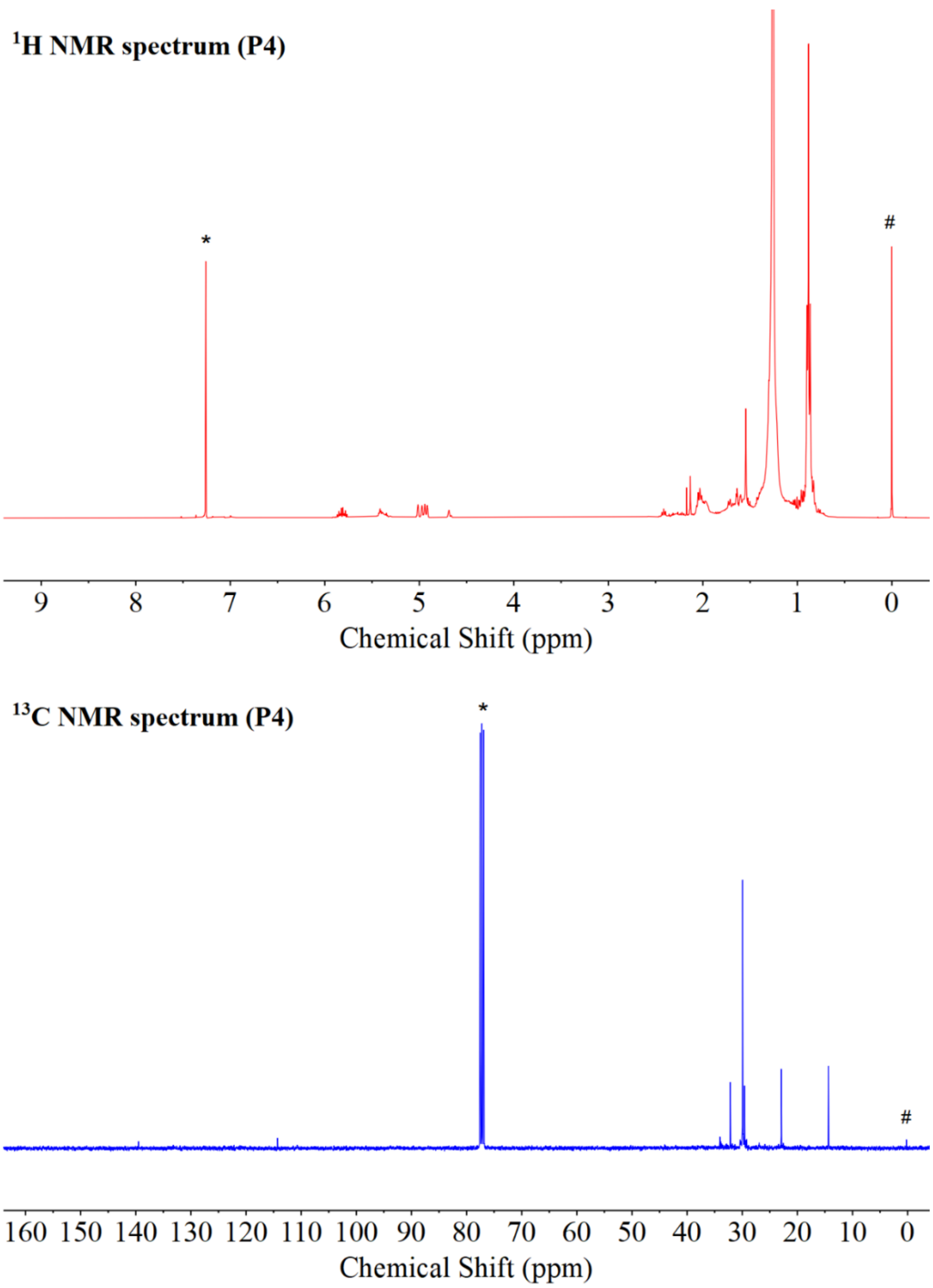

^{1}H NMR spectrum (P4)
*
#
9 8 7 6 5 4 3 2 1 0
Chemical Shift (ppm)
^{13}C NMR spectrum (P4)
*
#
160 150 140 130 120 110 100 90 80 70 60 50 40 30 20 10 0
Chemical Shift (ppm)

(c)

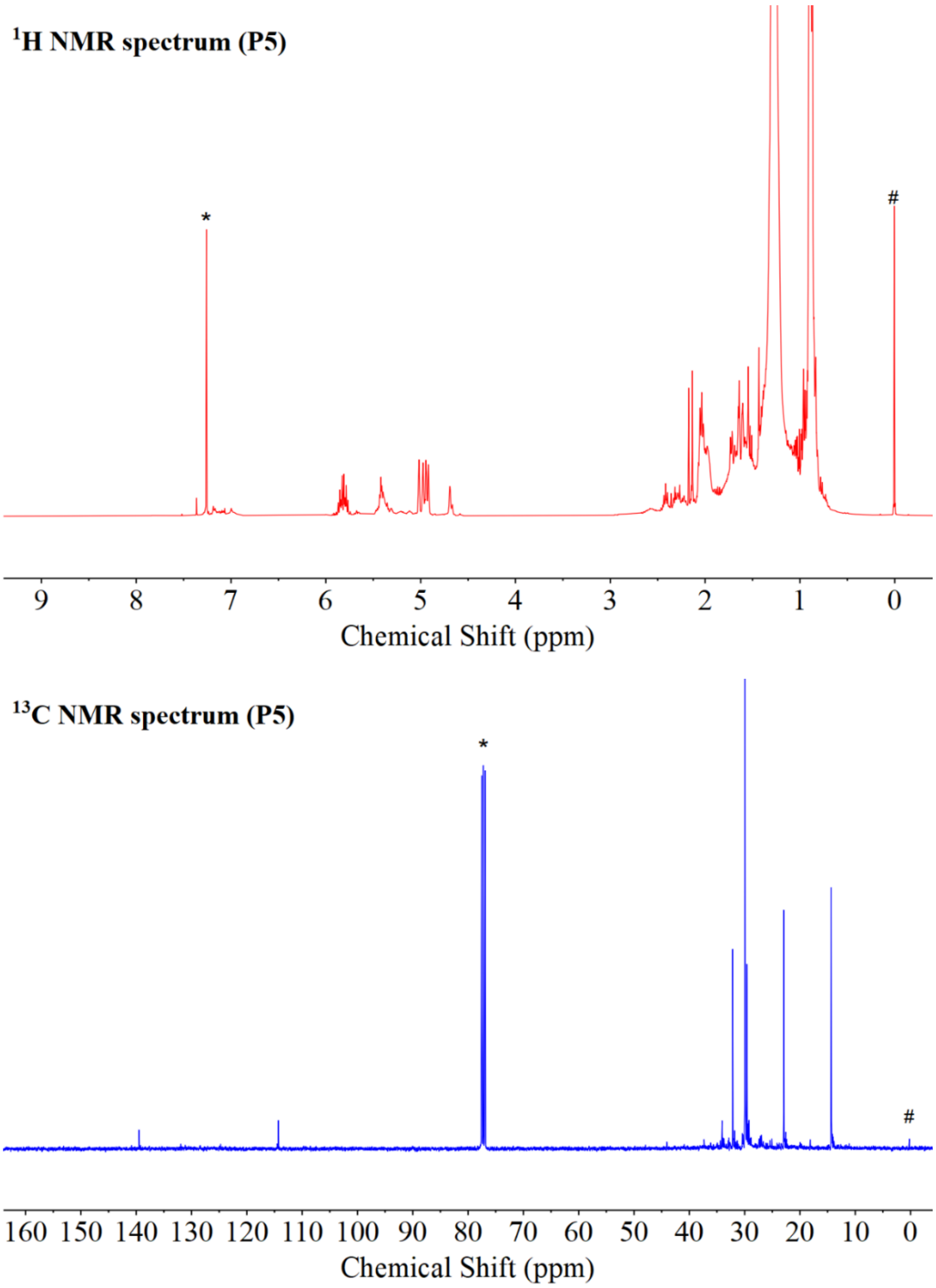

1H NMR spectrum (P5)
*
#
9 8 7 6 5 4 3 2 1 0
Chemical Shift (ppm)
13C NMR spectrum (P5)
*
#
160 150 140 130 120 110 100 90 80 70 60 50 40 30 20 10 0
Chemical Shift (ppm)

(d)

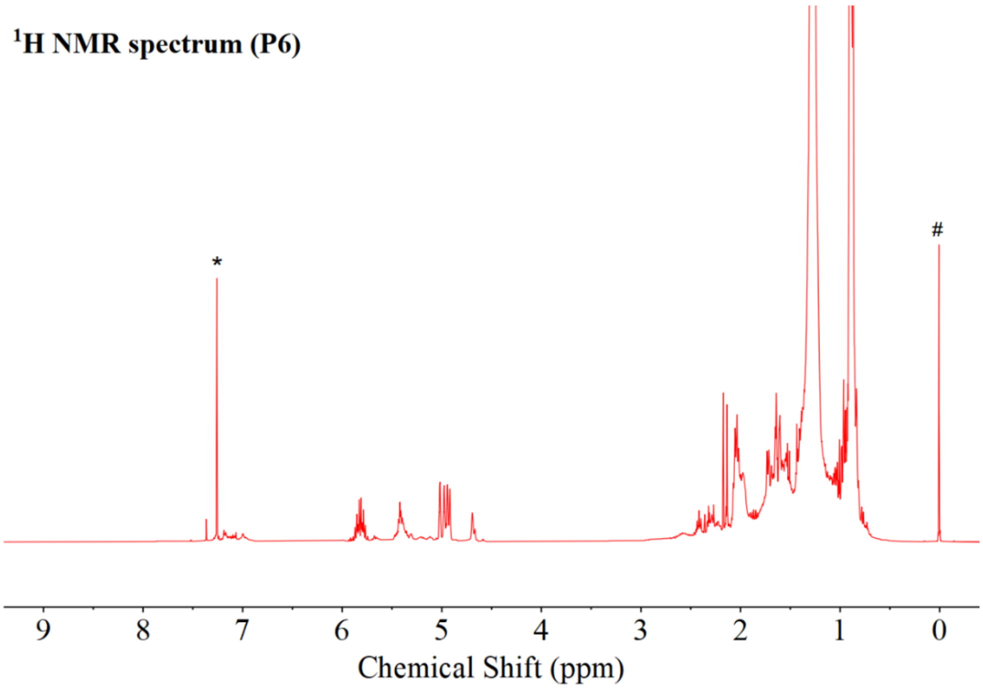

1H NMR spectrum (P6)
*
#
9 8 7 6 5 4 3 2 1 0
Chemical Shift (ppm)

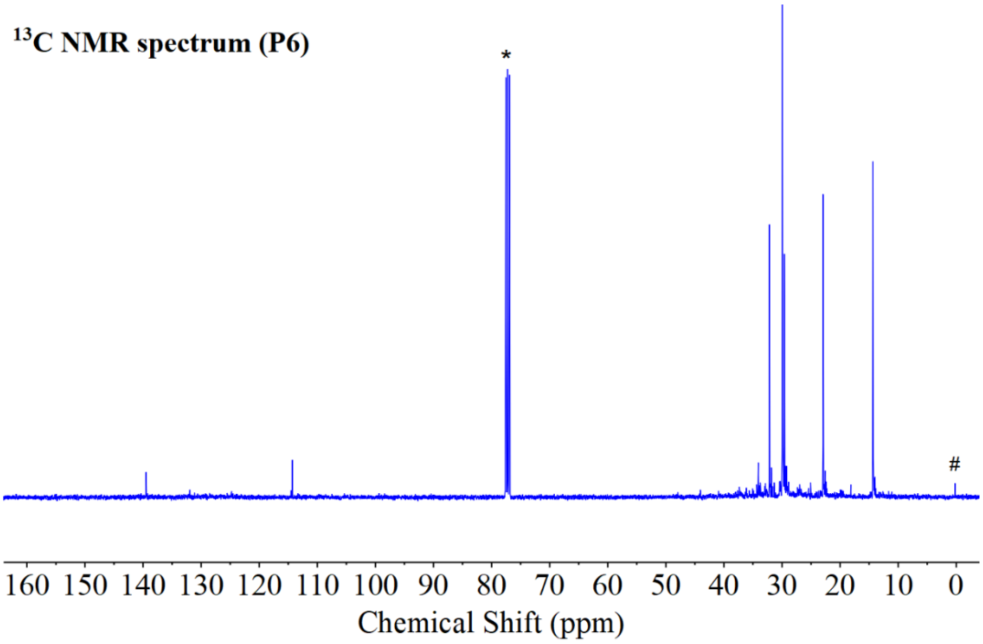

13C NMR spectrum (P6)
*
#
160 150 140 130 120 110 100 90 80 70 60 50 40 30 20 10 0
Chemical Shift (ppm)

(e)

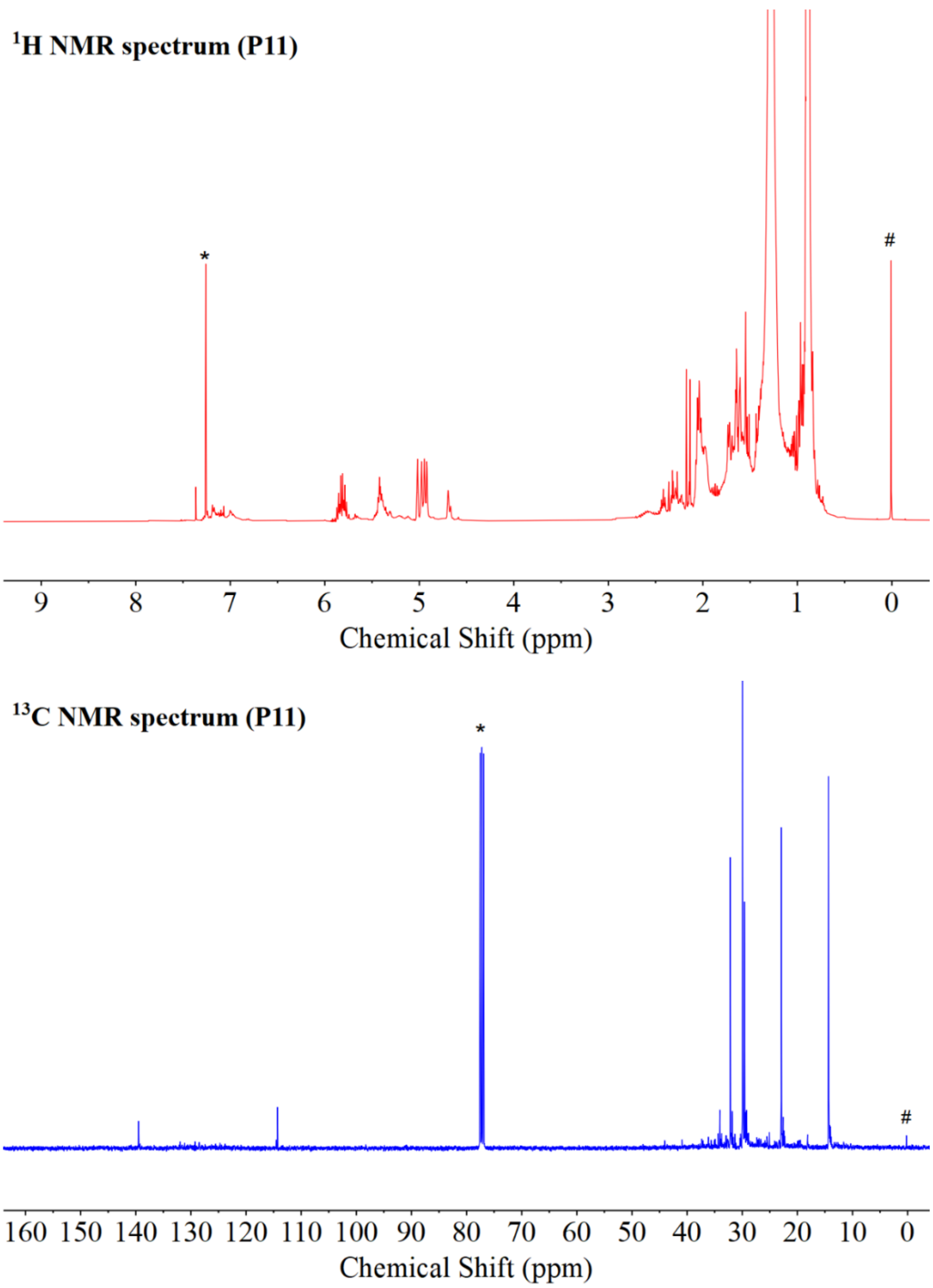

^{1}H NMR spectrum (P11)
*
#
9 8 7 6 5 4 3 2 1 0
Chemical Shift (ppm)
^{13}C NMR spectrum (P11)
*
#
160 150 140 130 120 110 100 90 80 70 60 50 40 30 20 10 0
Chemical Shift (ppm)

(f)

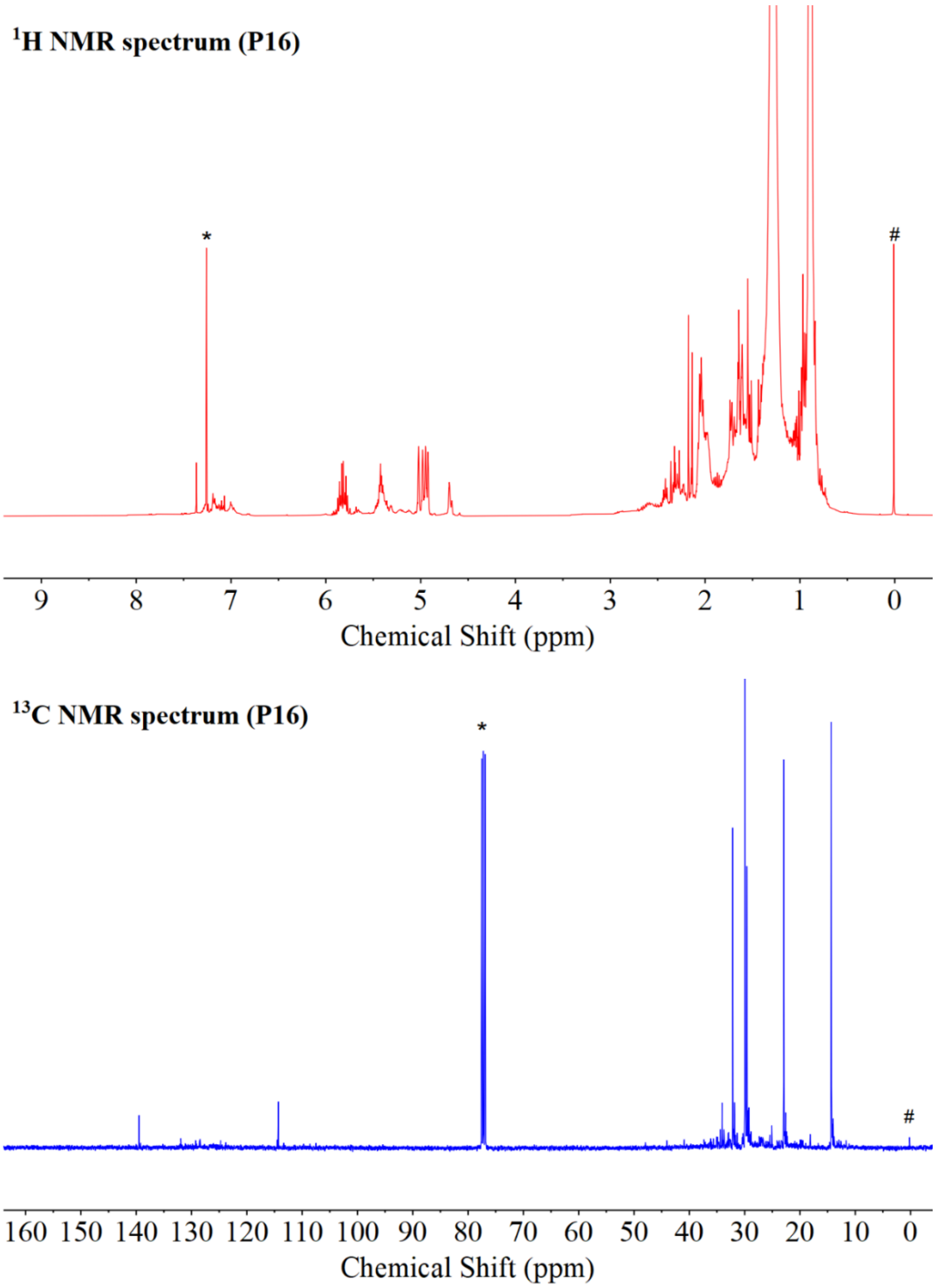

^{1}H NMR spectrum (P16)
*
#
9
8
7
6
5
4
3
2
1
0
Chemical Shift (ppm)
^{13}C NMR spectrum (P16)
*
#
160 150 140 130 120 110 100 90 80 70 60 50 40 30 20 10 0
Chemical Shift (ppm)

(g)

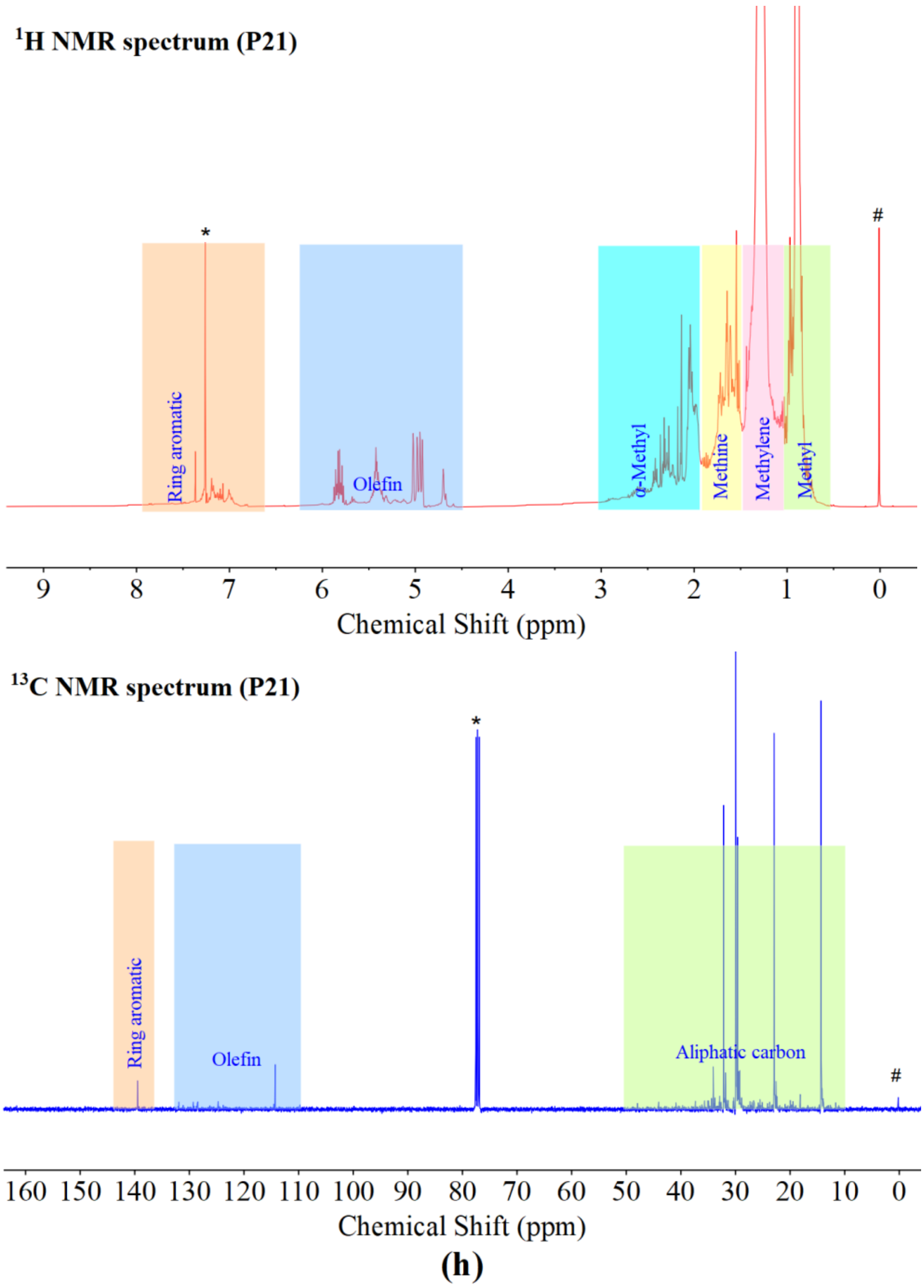

Figure S3. ^{1}H NMR spectra (red lines) and ^{13}C NMR spectra (bule lines) of the oil products obtained from different pressure experiments (listed in Table 1). * and # indicate the solvent (chloroform-D) and TMS, respectively.

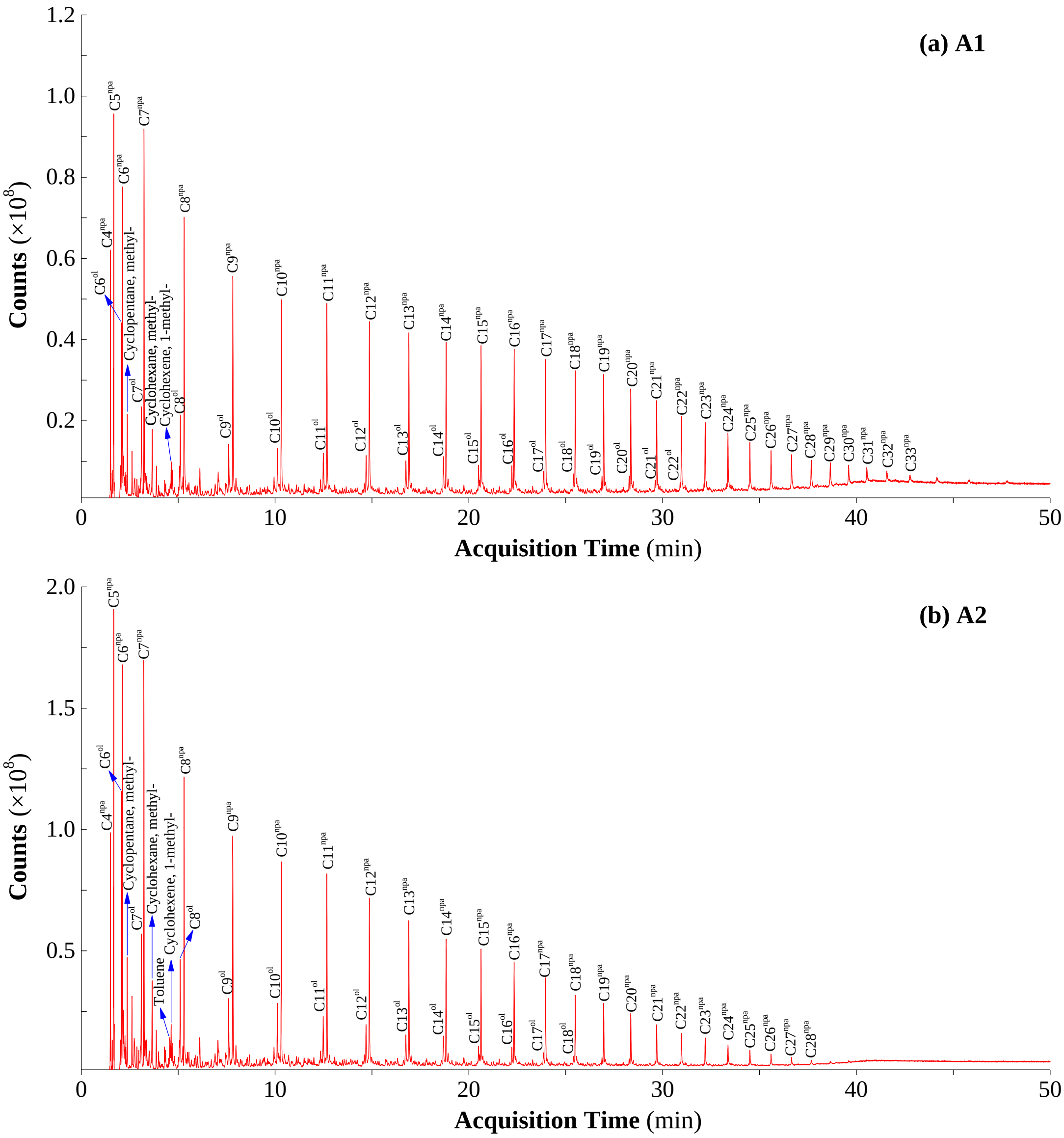

Figure S4. GC-MS chromatograms of the recovered oil samples from different atmosphere experiments (listed in Table 1).

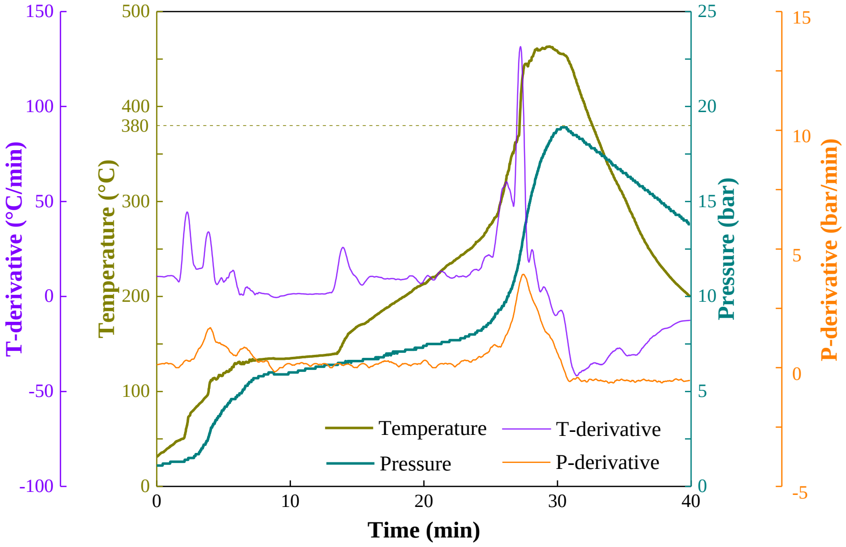
T-derivative (°C/min)
Temperature (°C)
Pressure (bar)
P-derivative (bar/min)
Time (min)
380
Temperature
T-derivative
Pressure
P-derivative

(a) L1

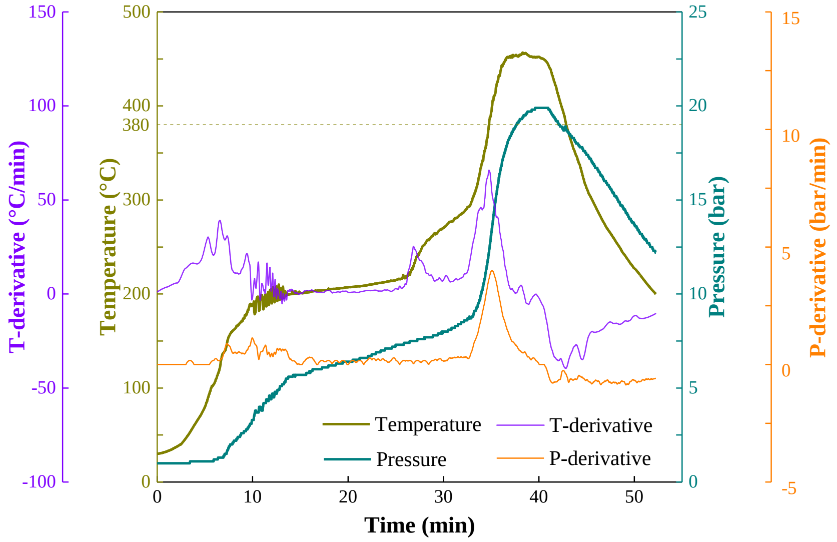
T-derivative (°C/min)
Temperature (°C)
Pressure (bar)
P-derivative (bar/min)
Time (min)
380
Temperature
T-derivative
Pressure
P-derivative

(b) L2

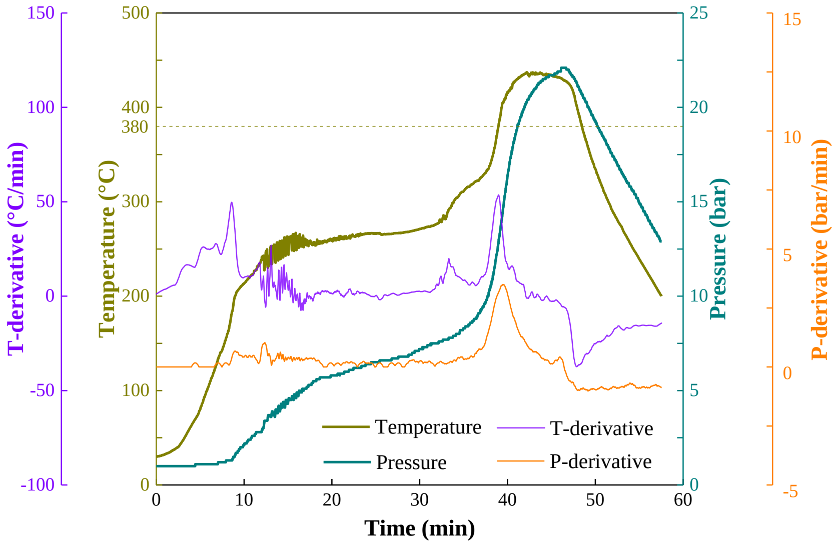
T-derivative (°C/min)
Temperature (°C)
Pressure (bar)
P-derivative (bar/min)
Time (min)
380
Temperature
T-derivative
Pressure
P-derivative

(c) L3

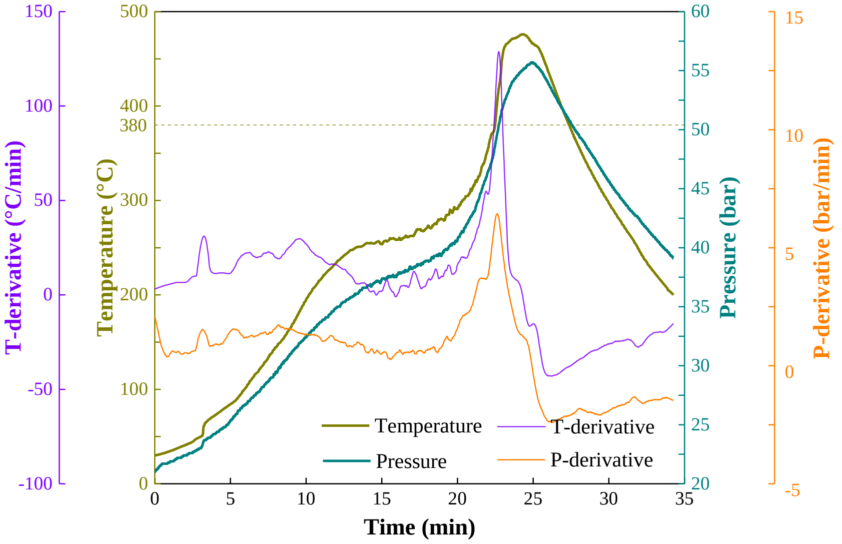
T-derivative (°C/min)
Temperature (°C)
Pressure (bar)
P-derivative (bar/min)
Time (min)
380
Temperature
T-derivative
Pressure
P-derivative

(d) L4

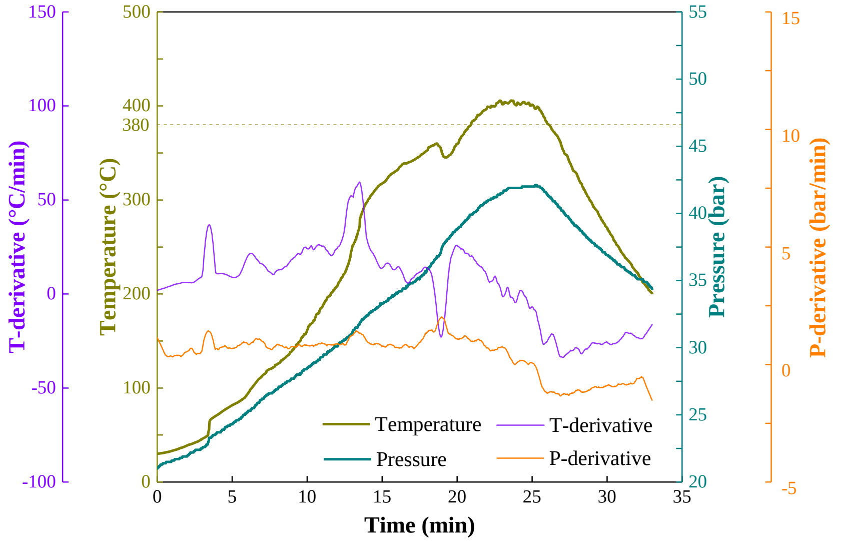
T-derivative (°C/min)
Temperature (°C)
Pressure (bar)
P-derivative (bar/min)
Time (min)
380
Temperature
T-derivative
Pressure
P-derivative

(e) L5

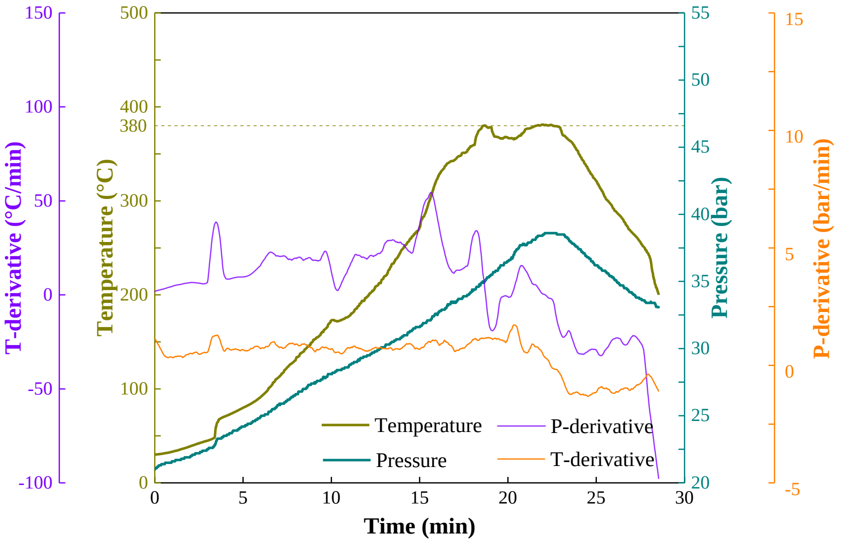
T-derivative (°C/min)
Temperature (°C)
Pressure (bar)
P-derivative (bar/min)
Time (min)
380
Temperature
P-derivative
Pressure
T-derivative

(f) L6

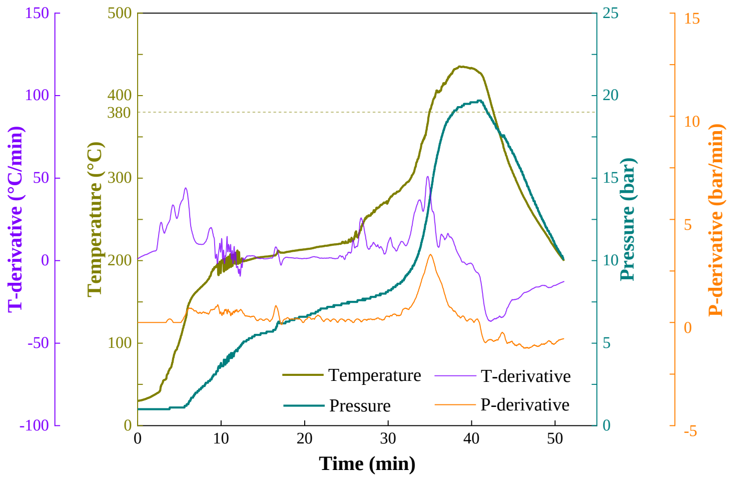
150
100
50
0
-50
-100
T-derivative (°C/min)
500
400
380
300
200
100
0
Temperature (°C)
25
20
15
10
5
0
Pressure (bar)
15
10
5
0
-5
P-derivative (bar/min)
0
10
20
30
40
50
Time (min)
Temperature
Pressure
T-derivative
P-derivative

(g) L7

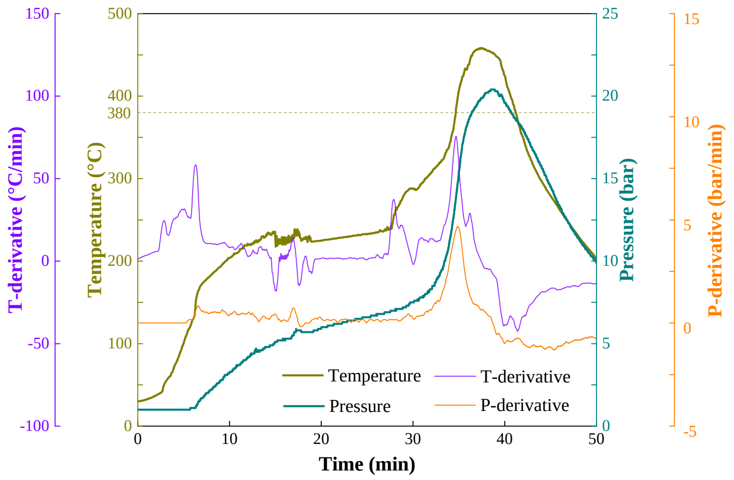
150
100
50
0
-50
-100
T-derivative (°C/min)
500
400
380
300
200
100
0
Temperature (°C)
25
20
15
10
5
0
Pressure (bar)
15
10
5
0
-5
P-derivative (bar/min)
0
10
20
30
40
50
Time (min)
Temperature
Pressure
T-derivative
P-derivative

(h) L8

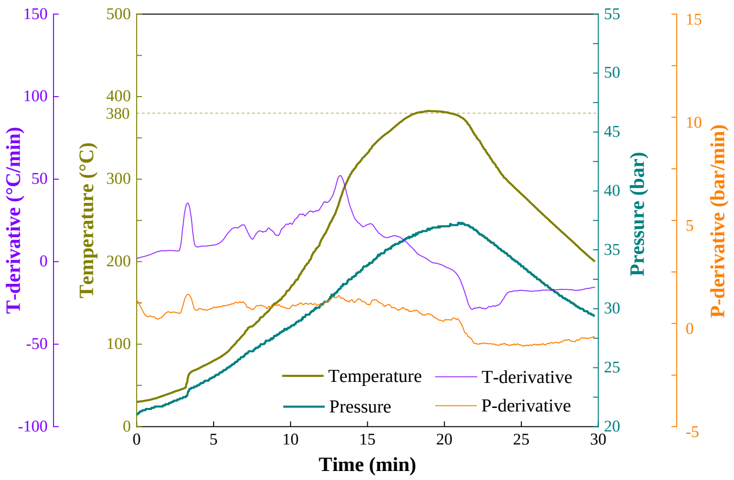
150
100
50
0
-50
-100
T-derivative (°C/min)
500
400
380
300
200
100
0
Temperature (°C)
55
50
45
40
35
30
25
20
Pressure (bar)
15
10
5
0
-5
P-derivative (bar/min)
0
5
10
15
20
25
30
Time (min)
Temperature
Pressure
T-derivative
P-derivative

(i) L9

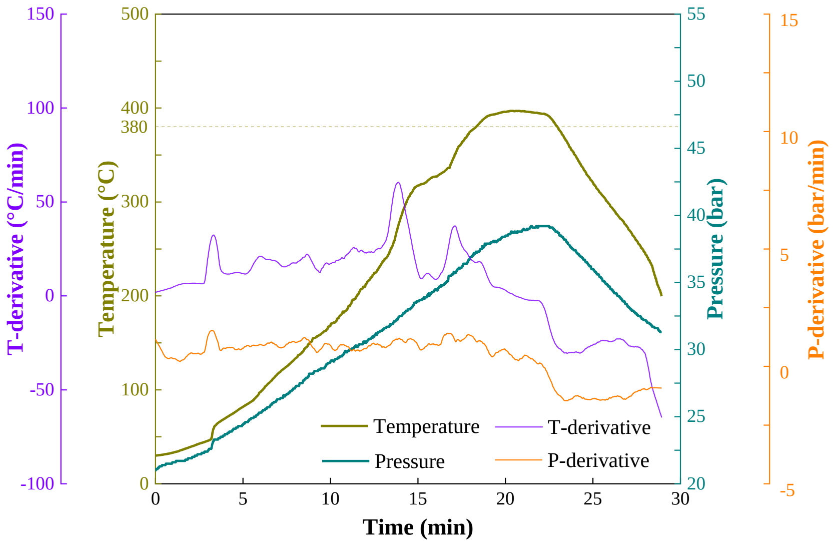

(j) L10

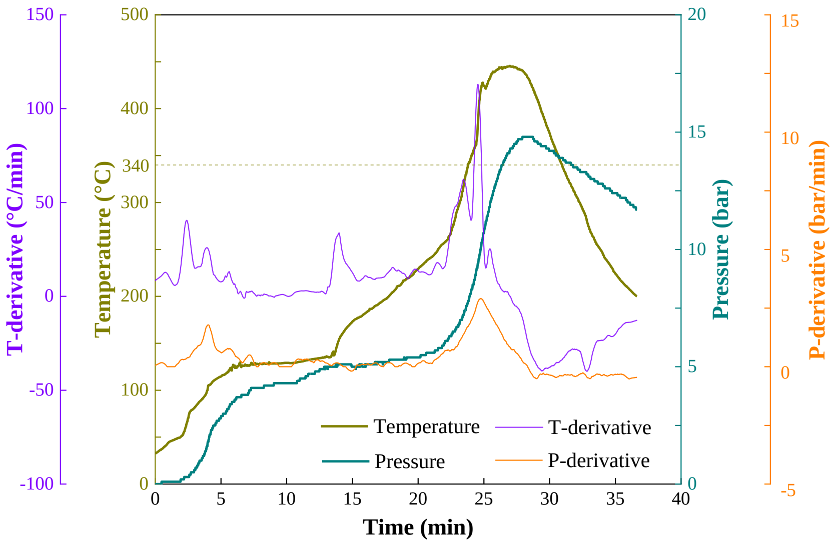

(k) L11

Figure S5. Temperature and pressure vs. time curves of experiments of adding liquid hydrocarbon (1-hexene, 1-octene, 1-decene, 1-octene or xylene) (listed in Table 1).

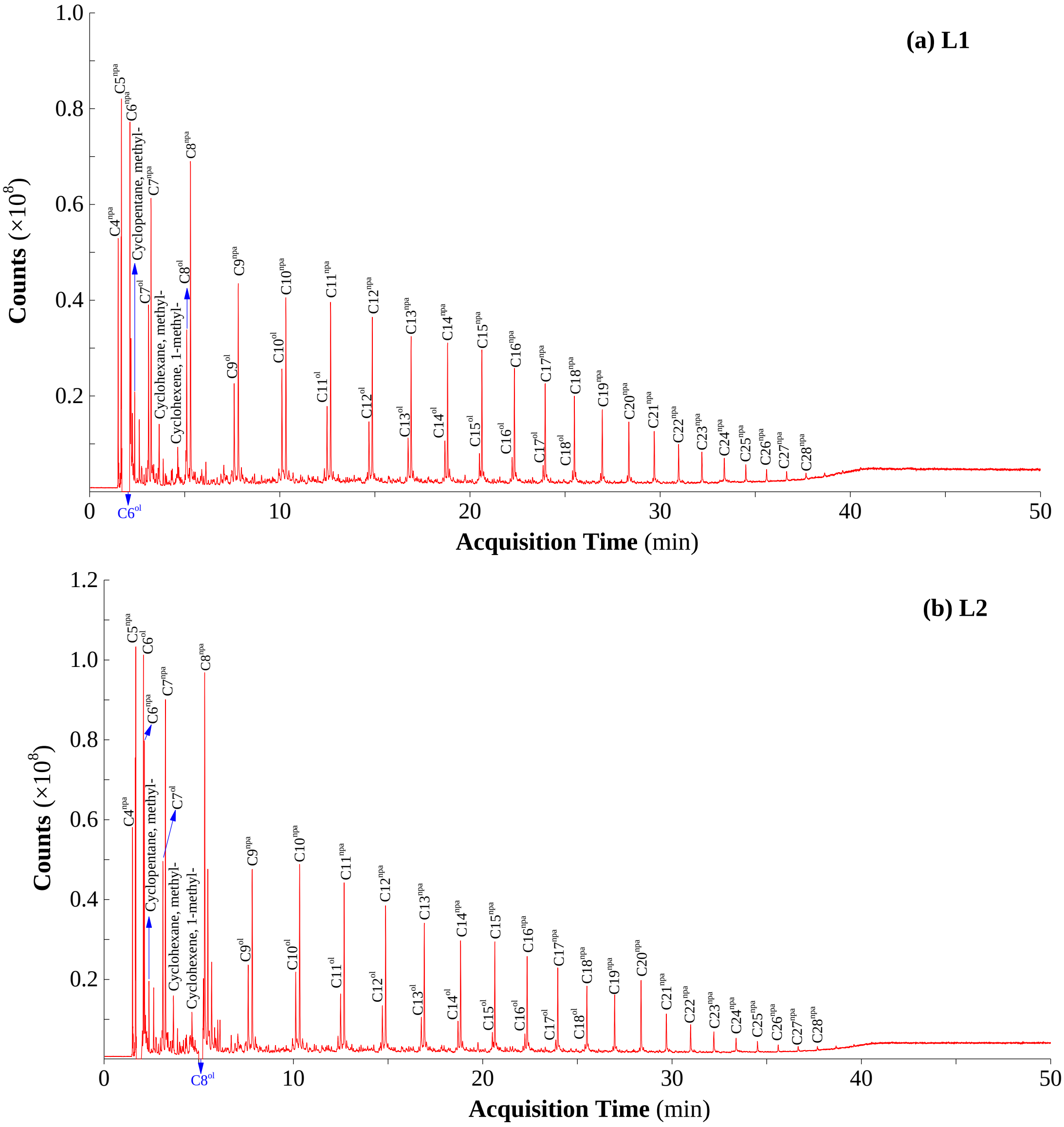

(a) L1
Counts (×10^8)
Acquisition Time (min)
1.0
0.8
0.6
0.4
0.2
0
10
20
30
40
50
$C4^{npa}$
$C5^{npa}$
$C6^{npa}$
$C6^{ol}$
Cyclopentane, methyl-
$C7^{ol}$
$C7^{npa}$
Cyclohexane, methyl-
Cyclohexene, 1-methyl-
$C8^{ol}$
$C8^{npa}$
$C9^{ol}$
$C9^{npa}$
$C10^{ol}$
$C10^{npa}$
$C11^{ol}$
$C11^{npa}$
$C12^{ol}$
$C12^{npa}$
$C13^{ol}$
$C13^{npa}$
$C14^{ol}$
$C14^{npa}$
$C15^{ol}$
$C15^{npa}$
$C16^{ol}$
$C16^{npa}$
$C17^{ol}$
$C17^{npa}$
$C18^{ol}$
$C18^{npa}$
$C19^{npa}$
$C20^{npa}$
$C21^{npa}$
$C22^{npa}$
$C23^{npa}$
$C24^{npa}$
$C25^{npa}$
$C26^{npa}$
$C27^{npa}$
$C28^{npa}$
(b) L2
Counts (×10^8)
Acquisition Time (min)
1.2
1.0
0.8
0.6
0.4
0.2
0
10
20
30
40
50
$C4^{npa}$
$C5^{npa}$
$C6^{ol}$
$C6^{npa}$
Cyclopentane, methyl-
$C7^{npa}$
$C7^{ol}$
Cyclohexane, methyl-
Cyclohexene, 1-methyl-
$C8^{ol}$
$C8^{npa}$
$C9^{ol}$
$C9^{npa}$
$C10^{ol}$
$C10^{npa}$
$C11^{ol}$
$C11^{npa}$
$C12^{ol}$
$C12^{npa}$
$C13^{ol}$
$C13^{npa}$
$C14^{ol}$
$C14^{npa}$
$C15^{ol}$
$C15^{npa}$
$C16^{ol}$
$C16^{npa}$
$C17^{ol}$
$C17^{npa}$
$C18^{ol}$
$C18^{npa}$
$C19^{npa}$
$C20^{npa}$
$C21^{npa}$
$C22^{npa}$
$C23^{npa}$
$C24^{npa}$
$C25^{npa}$
$C26^{npa}$
$C27^{npa}$
$C28^{npa}$

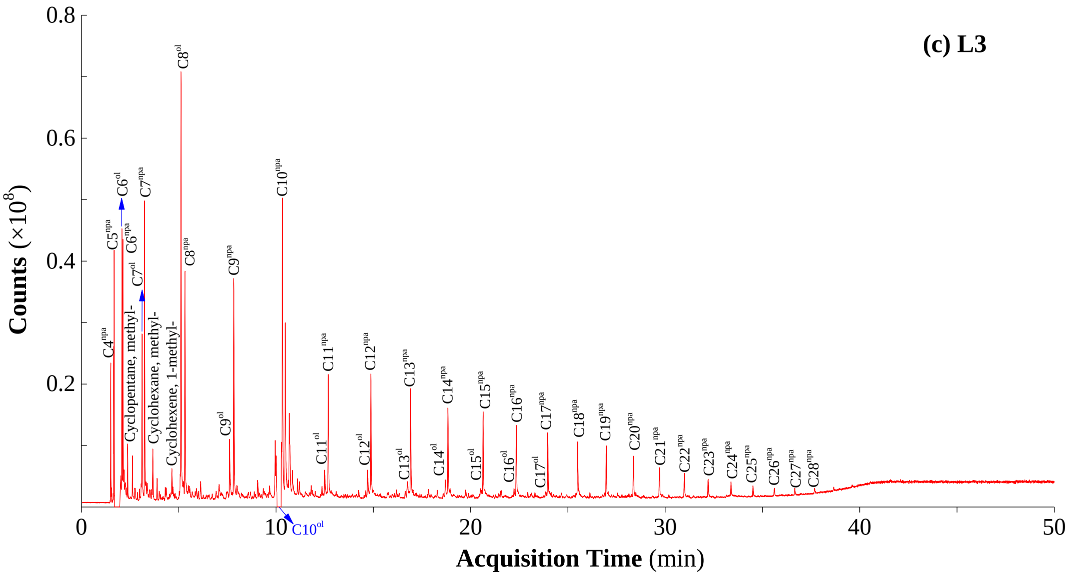
(c) L3
Counts (×10^8)
Acquisition Time (min)
Cyclopentane, methyl-
Cyclohexane, methyl-
Cyclohexene, 1-methyl-

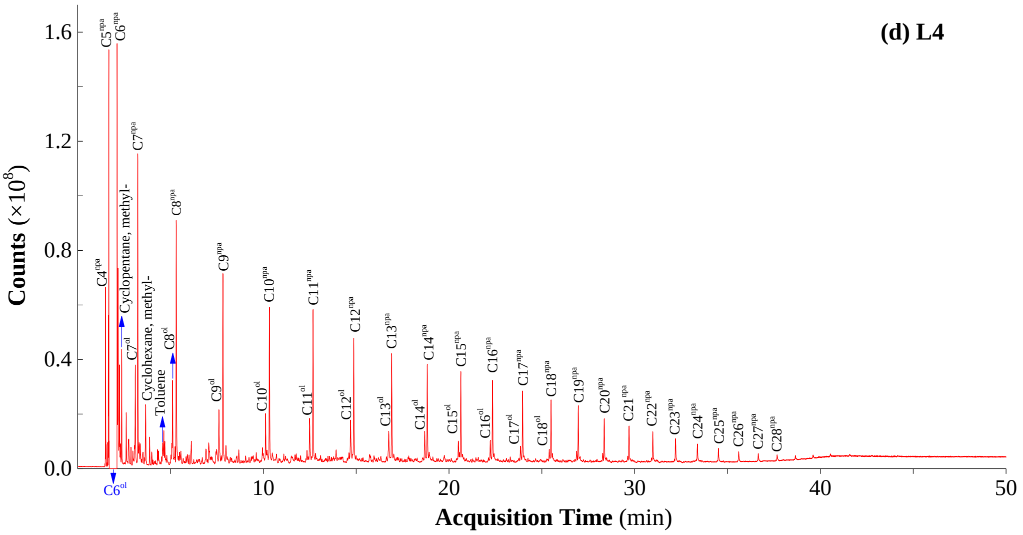
(d) L4
Counts (×10^8)
Acquisition Time (min)
Cyclopentane, methyl-
Cyclohexane, methyl-
Toluene

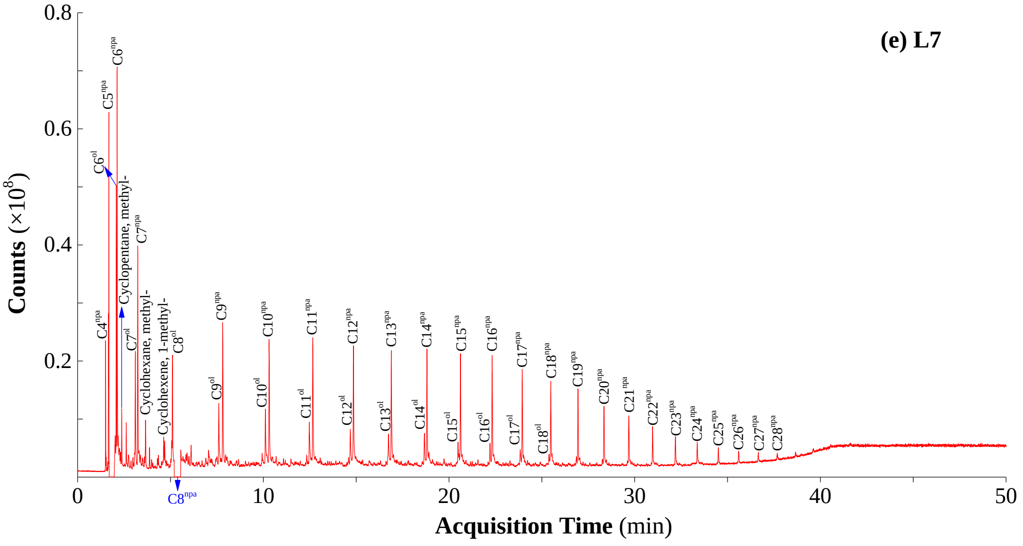
(e) L7
Counts (×10^8)
Acquisition Time (min)
Cyclopentane, methyl-
Cyclohexane, methyl-
Cyclohexene, 1-methyl-

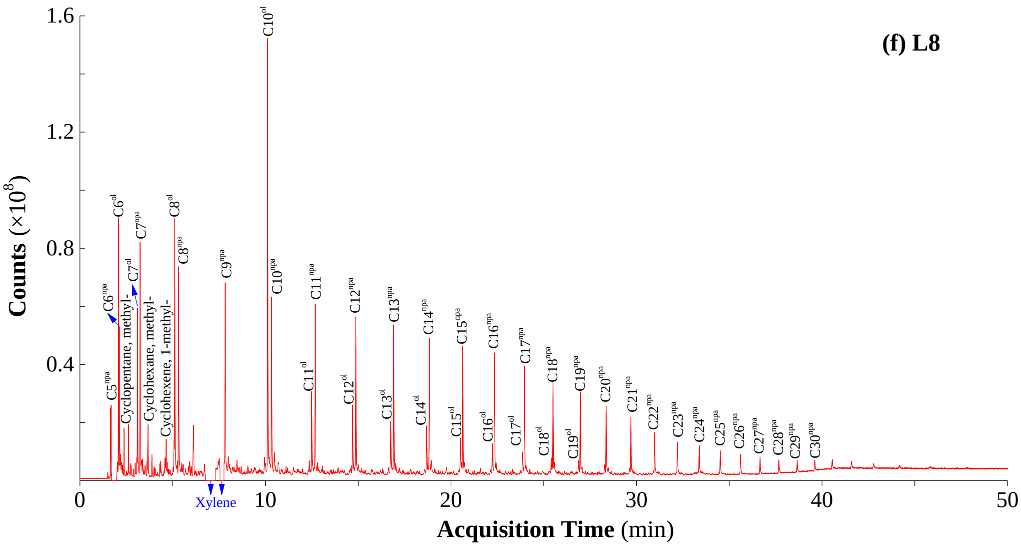

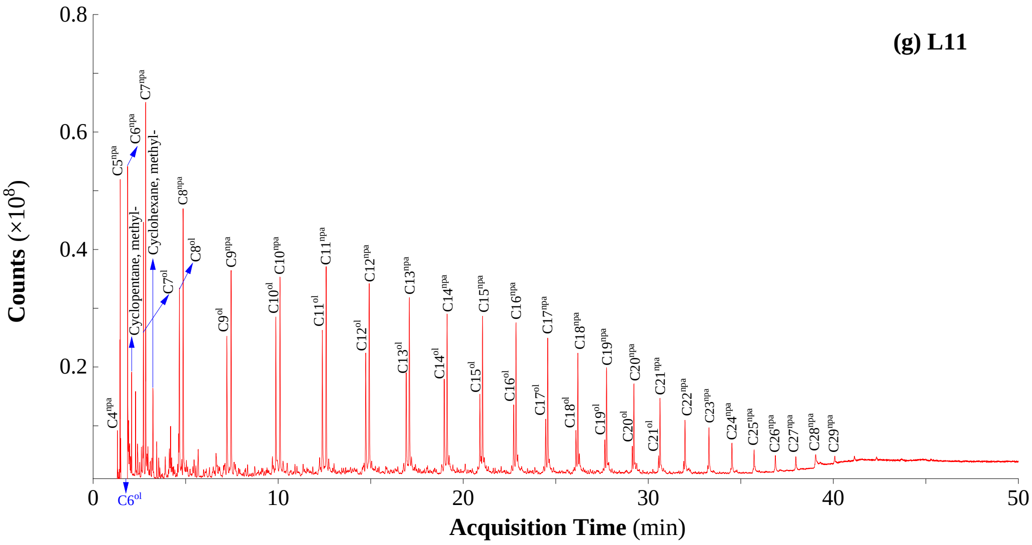

Figure S6. GC-MS chromatograms of the recovered liquid samples from experiments of adding liquid hydrocarbon (1-hexene, 1-octene, 1-decene, 1-octene or xylene) (listed in Table 1). Note that the peak of added liquid hydrocarbon is deducted.

Table S1. Different proton types and shift region from ^{1}H NMR spectra of liquid products [1,2].

Proton type	Chemical shift region (ppm)	Primary assignment of protons
Ring aromatic	6.6–8.0	Hydrogen atoms of aromatic rings
Olefin	4.5–6.5	Hydrogen atoms of olefin moieties
α-Methyl	2.0–3.0	CH_3 groups in the α-position to aromatic moieties
Methine	1.5–2.0	CH groups of paraffin chains and cyclanes
Methylene	1.0–1.5	CH_2 groups of paraffin chains and cyclanes
Methyl	0.6–1.0	CH_3 groups of saturated hydrocarbons

Reference

1. Sadykov, B.R.; Starikov, V.P.; Sadykov, R.K.; Kalabin, G.A. Determination of the fractional composition of merchantable oil using quantitative 1 H NMR spectra. *Pet. Chem.* **2012**, *52*, 22–27.
2. Morgenstern, M.; Cline, J.; Meyer, S.; Cataldo, S. Determination of the Kinetics of Biodiesel Production Using Proton Nuclear Magnetic Resonance Spectroscopy (1H NMR). *Energy Fuels* **2006**, *20*, 1350–1353.
